# Unlatching of the stem domains in the *Staphylococcus aureus* pore-forming leukocidin LukAB influences toxin oligomerization

**DOI:** 10.1016/j.jbc.2023.105321

**Published:** 2023-10-04

**Authors:** Juliana K. Ilmain, Sofya S. Perelman, Maria C. Panepinto, Irnov Irnov, Nicolas Coudray, Nora Samhadaneh, Alejandro Pironti, Beatrix Ueberheide, Damian C. Ekiert, Gira Bhabha, Victor J. Torres

**Affiliations:** 1 Department of Microbiology, New York University Grossman School of Medicine, New York, New York, USA; 2 Proteomics Laboratory, Division of Advanced Research Technologies, New York University Grossman School of Medicine, New York, New York, USA; 3 Applied Bioinformatics Laboratories, New York University Grossman School of Medicine, New York, New York, USA; 4 Department of Cell Biology, New York University Grossman School of Medicine, New York, New York, USA; 5 Antimicrobial-Resistant Pathogens Program, New York University Langone Health, New York, New York, USA; 6 Department of Biochemistry and Molecular Pharmacology, New York University Grossman School of Medicine, New York, New York, USA; 7 Department of Neurology, Center for Cognitive Neurology, New York University Grossman School of Medicine, New York, New York, USA; 8 Department of Host-Microbe Interactions, St Jude Children's Research Hospital, Memphis, Tennessee, USA

**Keywords:** *Staphylococcus aureus* (*S. aureus*), bacterial toxin, receptor, oligomerization, high-throughput screening

## Abstract

*Staphylococcus aureus* (*S. aureus*) is a serious global pathogen that causes a diverse range of invasive diseases. *S. aureus* utilizes a family of pore-forming toxins, known as bi-component leukocidins, to evade the host immune response and promote infection. Among these is LukAB (leukocidin A/leukocidin B), a toxin that assembles into an octameric β-barrel pore in the target cell membrane, resulting in host cell death. The established cellular receptor for LukAB is CD11b of the Mac-1 complex. Here, we show that hydrogen voltage-gated channel 1 is also required for the cytotoxicity of all major LukAB variants. We demonstrate that while each receptor is sufficient to recruit LukAB to the plasma membrane, both receptors are required for maximal lytic activity. Why LukAB requires two receptors, and how each of these receptors contributes to pore-formation remains unknown. To begin to resolve this, we performed an alanine scanning mutagenesis screen to identify mutations that allow LukAB to maintain cytotoxicity without CD11b. We discovered 30 mutations primarily localized in the stem domains of LukA and LukB that enable LukAB to exhibit full cytotoxicity in the absence of CD11b. Using crosslinking, electron microscopy, and hydroxyl radical protein footprinting, we show these mutations increase the solvent accessibility of the stem domain, priming LukAB for oligomerization. Together, our data support a model in which CD11b binding unlatches the membrane penetrating stem domains of LukAB, and this change in flexibility promotes toxin oligomerization.

*Staphylococcusaureus* (*S. aureus*) is an important global pathogen capable of causing infections in nearly all tissues (1). *S. aureus* lineages are categorized into clonal complexes (CC) based on genetic similarity at seven defined loci (2), and these lineages have adapted to thrive in different settings. CC8 *S. aureus* is one of the most extensively studied lineages, as it includes methicillin-resistant *S. aureus* responsible for the majority of skin and soft tissue infections in the United States (3, 4). Other lineages such as CC30 and CC45 *S. aureus* are associated with chronic infections (5, 6, 7, 8).

Among the virulence factors *S. aureus* employs to promote pathogenesis are a family of toxins known as the bi-component leukocidins (9). In human infections, *S. aureus* can produce up to five bi-component toxins: HlgAB, HlgCB, LukED, LukSF-PVL, and LukAB (also known as LukGH (10, 11)) (9). Together these toxins can target a variety of cells including leukocytes (9), erythrocytes (12, 13), and endothelial cells (14). Cell tropism of each leukocidin is determined by the presence of specific receptors on the host cell (15).

LukAB, one of three bi-component leukocidins encoded in the core *S. aureus* genome, is thought to be a major contributor to pathogenesis due to its potent action against phagocytes (10, 11, 16, 17, 18, 19, 20). LukAB is secreted as a stable heterodimer composed of LukA and LukB (21), with a final lytic state thought to consist of a membrane-embedded octameric β-barrel pore (22). The structure of LukAB, as well as other *S. aureus* leukocidins, can be divided into three domains: the rim, cap, and stem (23) (Fig. 1*A*). The rim domain consists of loops that come in close contact with cellular receptors on the plasma membrane of the target cell (15). In the dimeric soluble state, the stem domains of LukA and LukB are folded up against the cap domain (24). Upon oligomerization, *via* a mechanism not well understood, the stem domain is thought to undergo a conformational change that releases it from the cap domain, followed by its insertion into the host cell membrane, where it forms a β-barrel pore (22, 25, 26).

**Figure 1:**
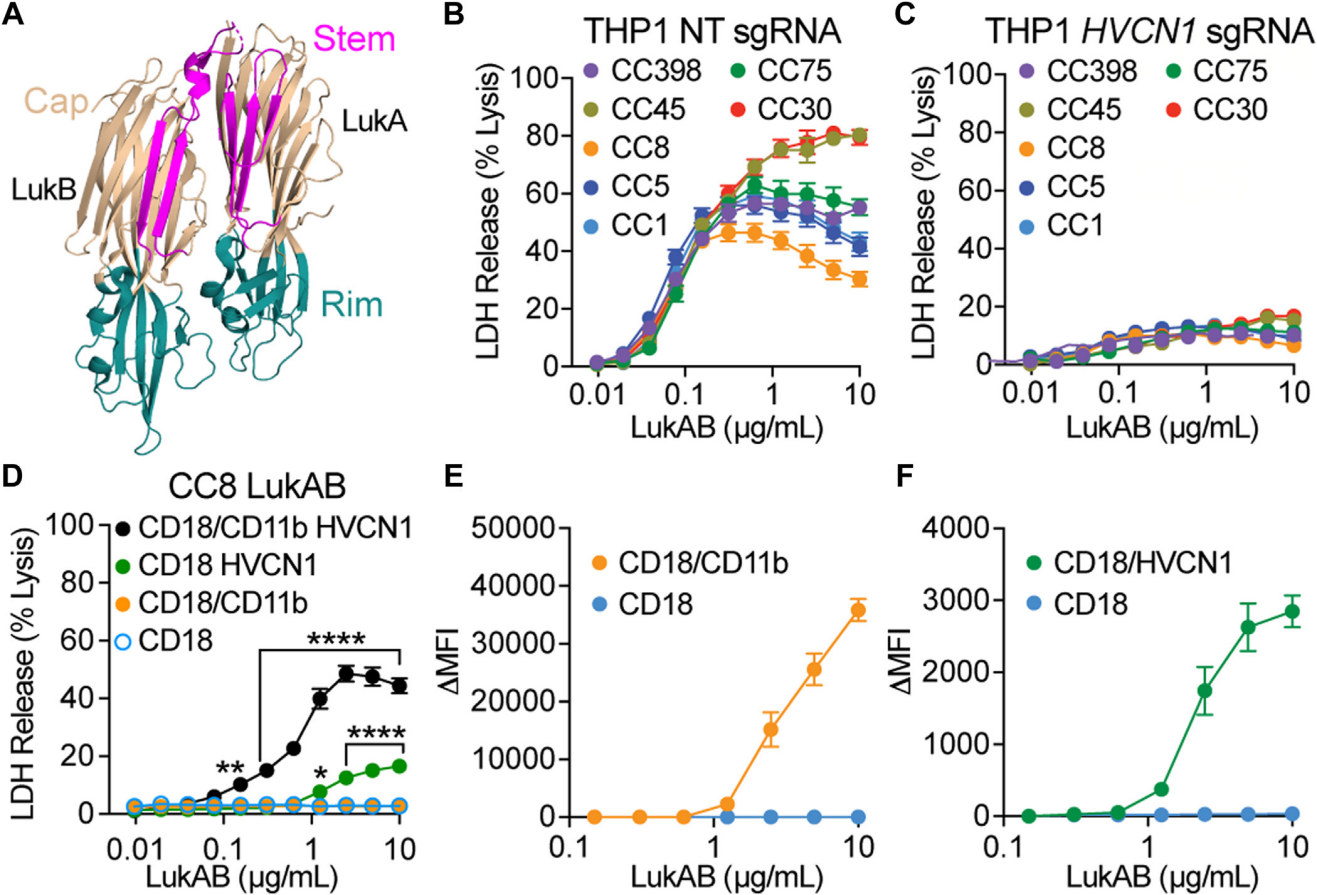
**HVCN1 is required for LukAB-mediated cell death.***A*, a schematic of LukAB depicting the rim, cap and stem domains (PDB 5k59 (24)). The cap domain is colored in *beige*, the rim domain in *teal*, and the stem domain in *magenta*. LukA and LukB are labeled. *B*, intoxication of THP-1 transduced with LentiCRISPRv2 expressing non-targeting (NT) sgRNA with LukAB allelic variants. Cell death measured by LDH release. Error bars represent SEM; N = 4. *C*, intoxication of THP-1 transduced with LentiCRISPRv2 expressing *HVCN1* sgRNA with LukAB allelic variants. Cell death measured by LDH release. Error bars represent SEM; N = 4. *D*, intoxication of CHO cells stably transduced with CD18, CD18/CD11b, CD18/HVCN1, or CD18/CD11b HVCN1 with CC8 LukAB. Cell death measured by LDH release. Error bars represent SEM. Statistical significance was determined by two-way ANOVA with Dunnett’s multiple comparisons test to CD18 CHO (∗∗∗∗*p* ≤ 0.001; ∗∗*p* = 0.0021; ∗*p* = 0.0363; no stars = not significant). Data is representative of six to eight independent experiments using six different protein preps for an N = 15 to 30. *E*, binding of CC8 LukAB to CHO cells stably transduced with CD18 shown as a control, and CD18/CD11b measured by Δ Median Fluorescence Intensity (ΔMFI) with PBS signal subtracted. Bound LukAB was detected with an anti-His PE-conjugated antibody by flow cytometry. Error bars represent SEM. Data is representative of six independent experiments using two different protein preps for an N = 12. *F*, binding of CC8 LukAB to CHO cells stably transduced with CD18 shown as a control, and CD18/HVCN1 measured by ΔMFI with PBS signal subtracted. Bound LukAB was detected with an anti-His PE-conjugated antibody by flow cytometry. Error bars represent SEM. Data is representative of six independent experiments using two different protein preps for an N = 12.

To initiate oligomerization and pore formation on the host cell, LukAB engages host receptors. The established cellular receptor for LukAB is CD11b (27), a subunit of the Mac-1 integrin that consists of CD18 and CD11b. LukAB specifically interacts with the I-domain of CD11b (17, 27, 28), with the primary binding site within the cap domain of LukA (21, 28). Recently, hydrogen voltage-gated channel 1 (HVCN1) was identified as a receptor for allelic variants CC30 and CC45 LukAB (16), but it was unknown if any other natural LukAB variants require HVCN1 for cytotoxic activity. Here, we show that all major LukAB variants require HVCN1 as a receptor in addition to CD11b. To understand the role of CD11b in the LukAB pore-forming process, we intoxicated CD11b-deficient cells with an alanine scanning library of CC8 LukAB to identify mutations that enable LukAB to exhibit full cytotoxicity in the absence of CD11b. We identified a set of residues that lie at the interface between the stem domain and the cap domain which, when mutated to an alanine, allow LukAB to kill cells in a CD11b-independent manner. We show that these mutations cause an increase in the solvent accessibility of the stem domain which changes the propensity for toxin oligomerization. These data suggest that the alanine mutations alter the conformation of the LukAB stem domain to mimic the state of LukAB post-CD11b binding, explaining the ability of the alanine variants to act independently of CD11b. Altogether, this study highlights how the conformation of the LukAB stem domain promotes toxin oligomerization and drives pore-formation.

## Results

### HVCN1 is required for the lytic activity of LukAB

One of the many features that render LukAB unique among other *S. aureus* bi-component leukocidins is its allelic diversity (10, 22). Previous work has shown that two variants, CC30 and CC45 LukAB, can function independent of the canonical LukAB receptor, CD11b, and instead can use HVCN1 as the sole receptor for cell targeting and killing (16). To evaluate if the lytic activity of other LukAB variants is dependent on HVCN1, we intoxicated THP-1 cells, which express both CD18/CD11b and HVCN1, transduced with Cas9 and either non-targeting (control) (Fig. 1*B*) or an *HVCN1* targeting sgRNA (Fig. 1*C*). While all LukAB variants killed THP-1 cells in a dose-dependent and saturable manner (Fig. 1*B*), *HVCN1* knockout THP-1 cells were resistant to LukAB-mediated cytotoxicity (Fig. 1*C*). These data show that HVCN1 is not only used by CC30 and CC45 LukAB, but it is required for the cytotoxicity of all LukAB allelic variants tested representing the major *S. aureus* clonal complexes.

To determine the contribution of each receptor to CC8 LukAB cytotoxicity and cell recruitment, we used CHO cells, a LukAB-resistant cell line that does not express either receptor. CHO cells were stably transduced with CD18 (used as a control throughout, as CD18 is required for CD11b to be stably localized on the cell surface (29)), CD18/CD11b, CD18/HVCN1 and both CD18/CD11b and HVCN1. Intoxication of these cell lines with LukAB shows minimal background activity with CD18 alone or CD18/CD11b, measurable cytotoxicity with HVCN1, and maximum activity with both CD18/CD11b and HVCN1 (Fig. 1*D*). Thus, both receptors are required for CC8 LukAB to reach optimal lytic activity. Notably, HVCN1 appears to be the more critical receptor, as we observe no cell death without it, while LukAB can exhibit modest activity without CD11b. Next, we tested the binding of LukAB to cells expressing the individual receptors. These data reveal that each receptor is sufficient to recruit the soluble LukAB dimer to the plasma membrane (Figs. 1, *E* and *F* and S1). Considering that LukAB can bind each receptor independently but that both receptors are required for full cytotoxicity, this suggests a role for each receptor that is beyond simply recruiting LukAB to its target cell.

### Alanine scanning screen identifies a region linked to CD11b receptor dependence

To begin to tease apart the role each receptor plays in the process of LukAB pore formation, we set out to identify mutations in CC8 LukAB that would allow it to bypass the requirement for CD11b and exhibit full activity using only HVCN1. Those mutations would provide insight into how CD11b bridges the gap between the measurable, yet minimal, cytotoxicity with HVCN1 alone *versus* both receptors.

**Figure 2:**
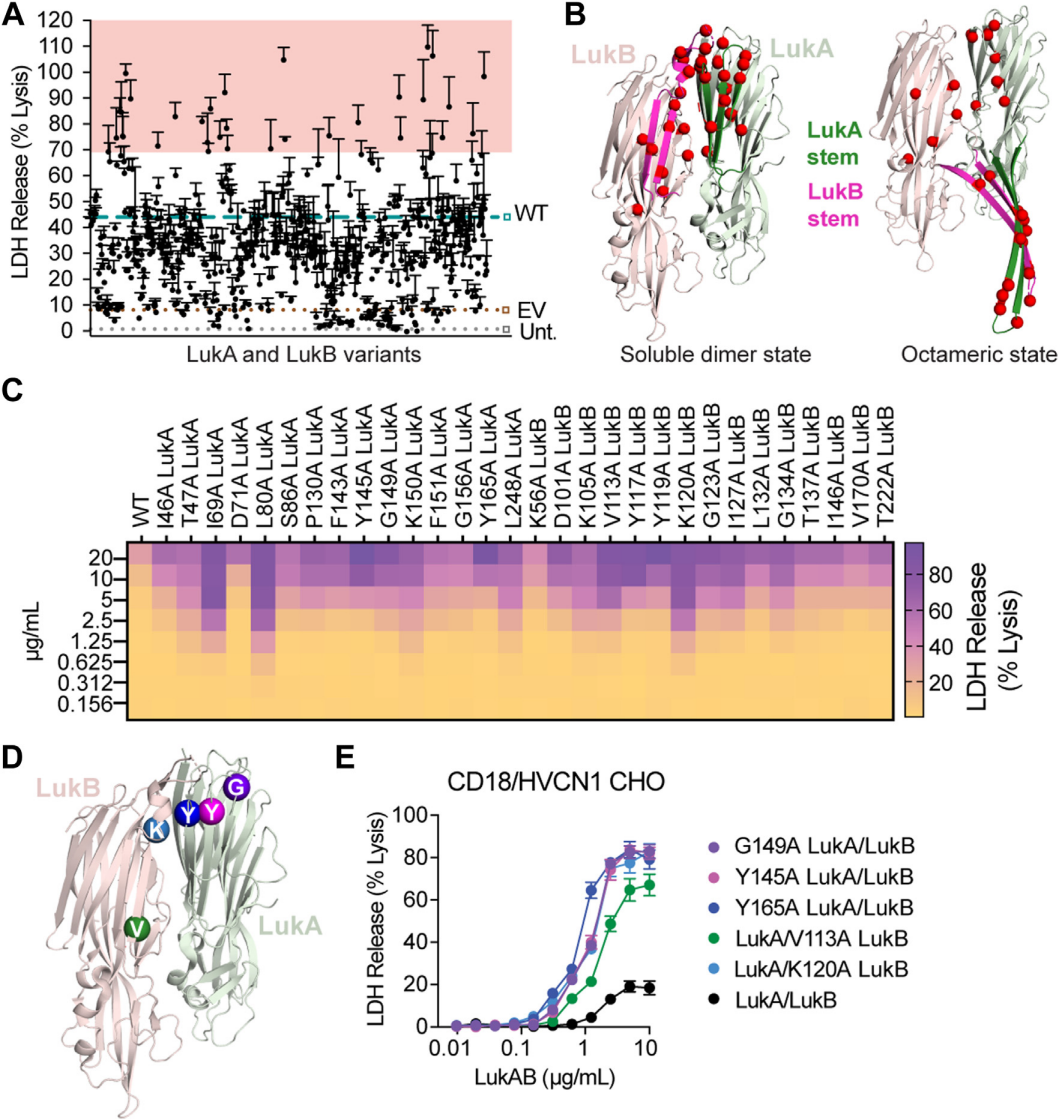
**Alanine scanning screen identifies mutations that allow CC8 LukAB to forgo dependency on CD11b.***A*, intoxication of CD18/HVCN1 CHO with LukAB-containing lysates. Each dot represents lysate from a different point mutant. WT LukAB, controls marked with *dashed lines* and *squares* outlined in *teal*, *brown*, and *gray*, respectively. LukAB-containing lysates that kill ≥ 70% of cells are shaded in *red*. Cell death measured by LDH release. Error bars represent SEM; N = 3. Also refer to Extended Data Figure S2. *B*, mapping of the top 30 residues (variants highlighted in *red* in Fig. 2*A*) on the LukAB structure that, when substituted for alanine, allow for enhanced CD11b-independent activity. *Left*, LukAB soluble dimer state (PDB 5k59 (24)); *right*, a dimer taken from the octameric state (PDB 4tw1 (22)). *Light green ribbon* = LukA, *dark green ribbon* = LukA stem domain. *Light pink ribbon* = LukB, *magenta ribbon* = LukB stem domain. Top 30 residues are highlighted with *red spheres*. *C*, heat map representation of the intoxication of CD18/HVCN1 CHO with crude purified LukAB variants that gained CD11b-independent cytolytic activity. Cell death measured by LDH release. Heat map shows the mean of N = 3 independent experiments. *D*, mapping of the mutated residues of select variants chosen for *S. aureus* expression, purification, and characterization on the dimeric structure (PDB 5k59 (24)). *Light green ribbon* = LukA, *light pink ribbon* = LukB. *Purple sphere* = G149 LukA, *pink sphere* = Y145 LukA, *navy blue sphere* = Y165 LukA, *green sphere* = V113 LukB, *light blue sphere* = K120 LukB. *E*, intoxication of CD18/HVCN1 CHO with purified WT and select LukAB variants. Cell death measured by LDH release. Error bars represent SEM; N = 3. EV, empty vector; Unt, untransformed.

We generated an alanine scanning mutant library of CC8 LukA and LukB optimized for *E. coli* expression, resulting in 615 constructs: 313 LukA mutants with wild-type (WT) LukB and 302 LukB mutants with a WT LukA. A 6×His tag on LukA was included for purification purposes. To detect LukA and LukB separately, we also included a cMyc tag on LukB, which did not significantly alter the cytotoxicity of LukAB (Fig. 2, Extended Data Fig. S1).

We screened for mutations that could enhance LukAB activity in the absence of CD11b by intoxicating CD18/HVCN1 CHO cells with *E. coli* lysates containing each LukAB alanine variant (Fig. 2*A*, Extended Data Fig. S2). While lysates were not normalized for toxin quantities, we assessed LukA and LukB presence by ELISA to verify toxin production (Fig. S3, Extended Data Fig. S2). Thirty toxins, 15 with alanine substitutions in LukA and 15 with alanine substitutions in LukB, were able to kill 70% of cells or more (Fig. 2*A*). In comparison, in this assay, WT LukAB exhibits saturable killing around 40%, suggesting these 30 alanine mutations allow LukAB to have improved cytotoxic activity in the absence of CD11b.

Strikingly, the mutations that allow for CD11b-independent activity cluster in one region: either on the stem domain or on the cap domain β strands facing the stem domain in the soluble dimer state (Fig. 2*B*), hereafter the stem/cap interface. This suggests a functional connection between this protein region and LukAB CD11b-dependence. To validate our screen, we performed an independent expression and crude purification of the top 30 hits (Fig. S4) and tested these proteins in a secondary screen on CD18/HVCN1 CHO (Fig. 2*C*). Although we detected a range in cytotoxicity, all variants exhibit cytotoxicity greater than WT LukAB (Fig. 2*C*). To investigate how mutations at the LukAB stem/cap interface allow for enhanced CD11b-independent activity, we selected five mutants with alanine substitutions that are positioned at different locations along the LukA and LukB stem domains for functional characterization: G149A LukA, Y145A LukA, Y165A LukA, V113A LukB, and K120A LukB (Fig. 2*D*), hereafter using proteins purified from *S. aureus* which were normalized for LukAB quantities (Fig. S5). Intoxication of CD18/HVCN1 CHO with these select variants demonstrates that alanine substitutions at the stem/cap interface of LukAB lead to enhanced CD11b-independent cytotoxicity (Fig. 2*E* and Table S1).

### Alanine mutations at the stem/cap interface provide a cytotoxic advantage to LukAB only in the absence of CD11b

**Figure 3:**
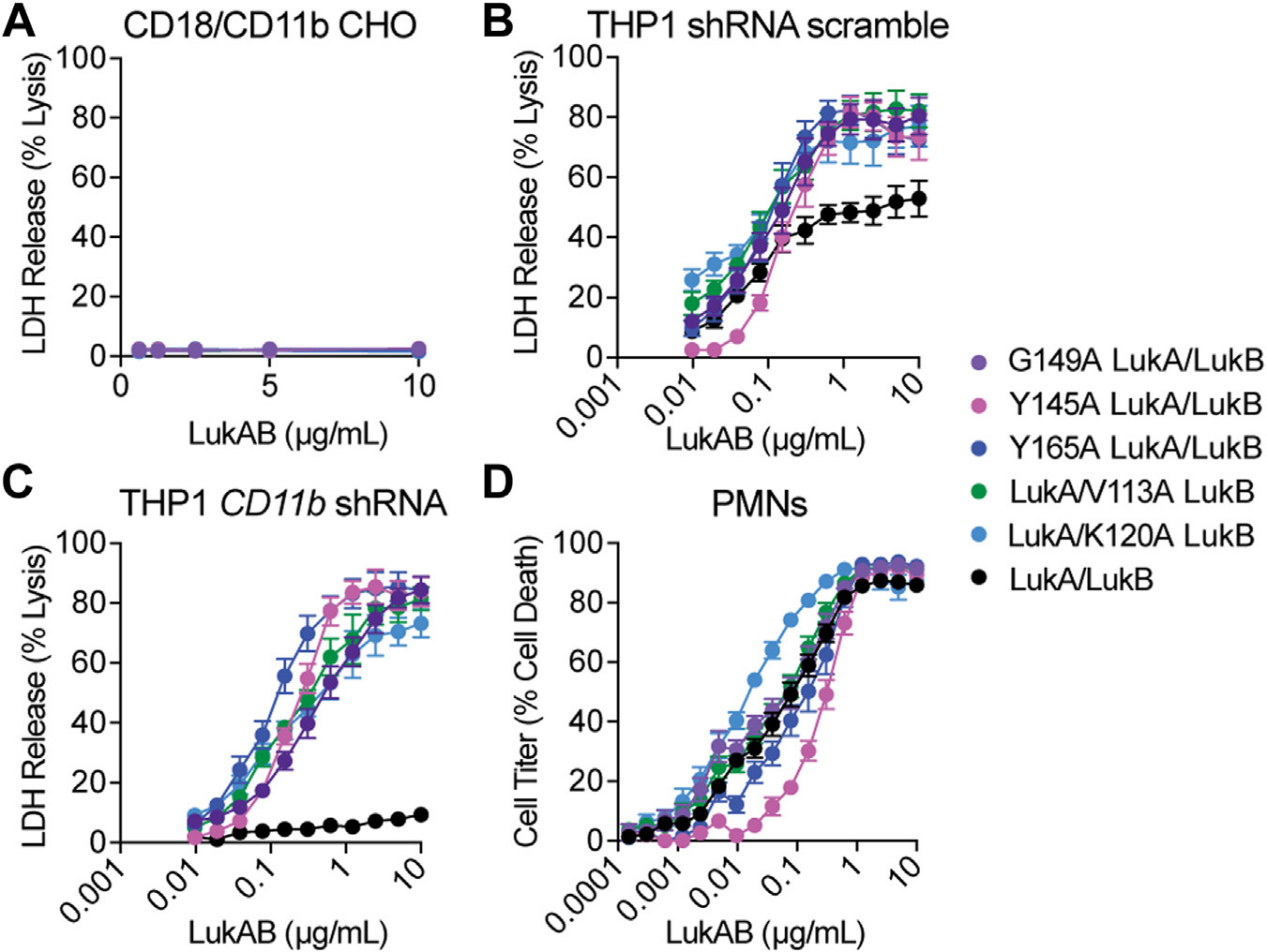
**Mutating residues at the stem/cap interface gives LukAB a cytotoxic advantage in the absence of CD11b.***A*, intoxication of CD18/CD11b CHO with purified WT and select LukAB variants. Cell death measured by LDH release. Error bars represent SEM; N = 4. *B*, intoxication of THP-1 scramble shRNA with purified WT and select LukAB variants. Cell death measured by LDH release. Error bars represent SEM; N = 4. *C*, intoxication of THP-1 *CD11b* shRNA with purified WT and select LukAB variants. Cell death measured by LDH release. Error bars represent SEM; N = 4. *D*, intoxication of primary human PMNs with purified WT and select LukAB variants. Cell death measured by CellTiter. Error bars represent SEM; N = 11 independent donors.

While these alanine variants display potent lytic activity in the absence of CD11b (Fig. 2*E*), we wanted to determine if the mutations at the stem/cap interface allow LukAB to bypass either receptor or both receptors. To test this, we intoxicated CD18 and CD18/CD11b CHO cells to determine whether the variants have enhanced activity in the absence of HVCN1. Consistent with the WT toxin, these alanine variants show no activity without HVCN1 (Figs. 3*A* and S6). Given the engineered CHO cells overexpressed each receptor, we verified this phenotype was not dependent on HVCN1 overexpression by evaluating the cytotoxicity of these variants on cells with native levels of HVCN1. We tested the cytotoxicity of the select alanine variants on THP-1 cells transduced with scramble (control) (Fig. 3*B*) or *CD11b* shRNA (Fig. 3*C*). Under scramble shRNA conditions, WT CC8 LukAB has saturable killing around 50% cell death (Fig. 3*B*), which has previously been observed (16, 19). Interestingly, the alanine variants exhibit saturable killing around 80% of cell death. More strikingly, when CD11b is depleted, the alanine variants maintain their full activity while the WT toxin is reduced to very minimal activity (Fig. 3*C*). These data establish that alanine mutations at the stem/cap interface allow CD11b-independent LukAB activity even with native levels of HVCN1.

LukAB is particularly potent toward human neutrophils (10, 11), which are often the first immune cells to encounter an infection (30). CD18/CD11b and HVCN1 are co-produced on phagocytes, allowing LukAB to target these critical innate immune cells (31, 32). Thus, primary human neutrophils (PMNs) were intoxicated to evaluate if these alanine variants have a cytotoxic advantage over WT on primary cells (Fig. 3*D*). LC50 values for G149A and V113A are comparable to WT, ∼5× less for K120A, and ∼2.5 to 5× greater for Y145A and Y165A (Table S2). Thus, collectively there is no major advantage of the alanine mutations for LukAB when targeting neutrophils.

### Alanine mutations at the stem/cap interface promote LukAB oligomerization

Curious about the functional consequence of the alanine mutations at the stem/cap interface, we sought to determine the mechanism allowing these LukAB variants to bypass CD11b. We initially speculated these mutations may enhance LukAB binding to HVCN1. To test this, we used Alexa Fluor 488 labeled LukAB (Fig. S7*A*) to detect binding to CD18/HVCN1 CHO cells. We first established that labeling did not impact toxin activity (Fig. S7*B*). While we observe very minimal background binding to CD18 CHO, we observe similar or weaker levels of binding of the alanine variants to CD18/HVCN1 CHO as compared to WT but not enhanced binding that might explain the CD11b-independent cytotoxicity (Fig. S7*C*). As a complementary approach, we also assessed binding by performing a pull-down assay using purified HVCN1. Relative to the amount of resin-bound HVCN1, the amount of LukAB bound to HVCN1 with the alanine variants is comparable to or slightly less than WT LukAB (Fig. S7*D*). Together these experiments suggest that the alanine mutations at the stem/cap interface do not allow LukAB to bypass CD11b because of improved HVCN1 binding.

**Figure 4:**
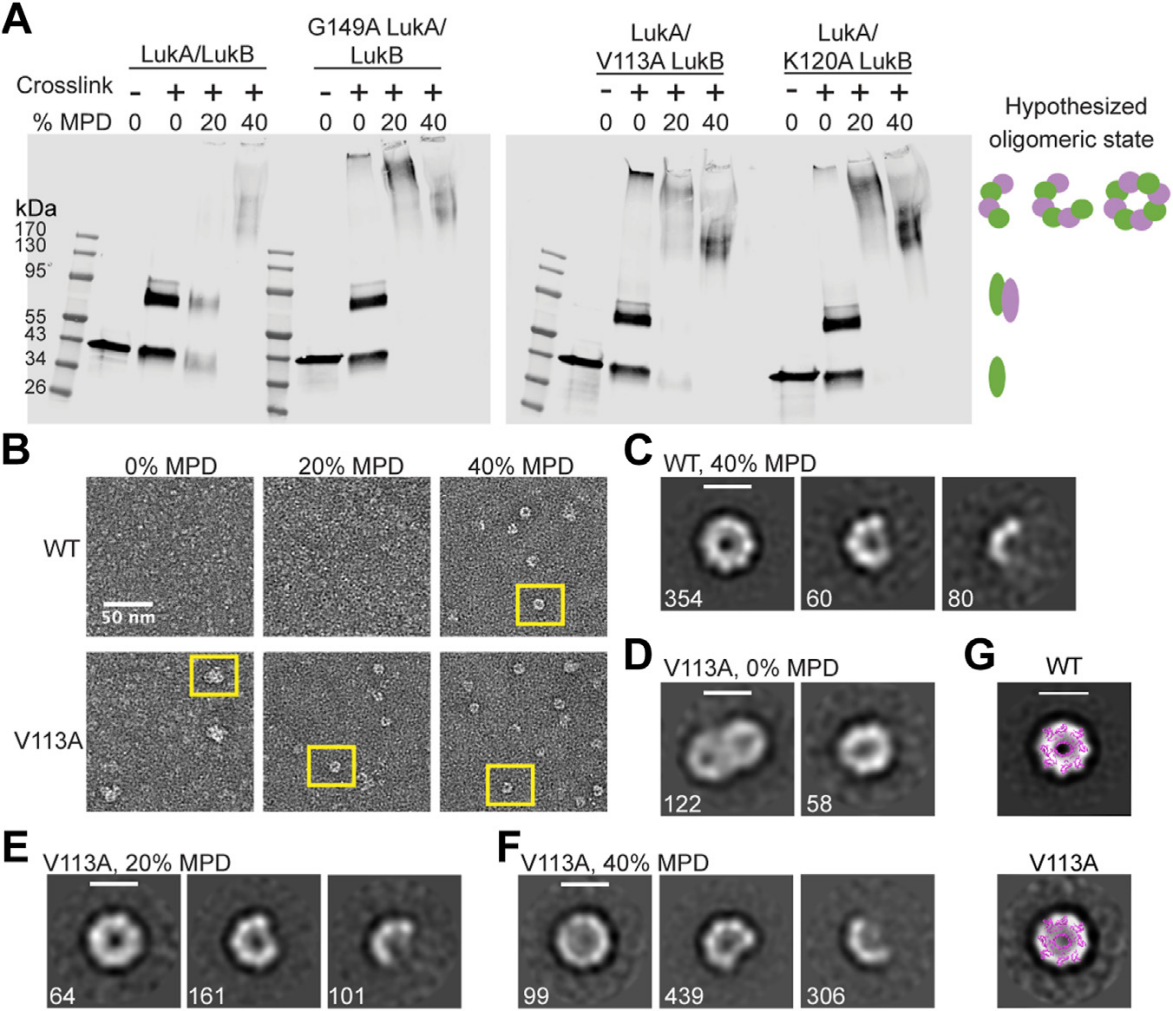
**Disrupting the interface at the LukAB stem domain promotes LukAB to oligomerize more readily.***A*, Western blots of WT, G149A, K120A, and V113A LukAB un-crosslinked with no additive, crosslinked with no additive, crosslinked with 20% MPD, or crosslinked with 40% MPD. LukAB was detected with anti-His antibody. Schematic on the *right* represents predicted LukAB oligomeric states. Images are representative of N = 3 independent blots. *B*, negative stain electron micrographs of WT and V113A LukAB in 0% MPD, 20% MPD or 40% MPD. Scale bar is 50 nm. Examples of LukAB oligomers are boxed in yellow. *C*–*F*, 2D class averages of WT LukAB in 40% MPD (*C*), V113A LukAB in 0% MPD (*D*), V113A LukAB in 20% MPD (*E*), and V113A LukAB in 40% MPD (*F*), labeled with the number of particles in each class. Scale bar is 100 Å. G. Overlay of the LukAB octamer (PDB 4tw1 (22)) with negative stain 2D class averages for WT and V113A shows that circular-shaped negative stain classes are consistent in size and shape with a LukAB octamer.

Having ruled out enhanced HVCN1 binding as a mechanistic explanation for the alanine variants, we next explored enhanced oligomerization as a potential mechanism. Previous studies have shown that bi-component leukocidins assemble into a pre-pore prior to forming a membrane-penetrating pore, which is a result of stem domains partially unfolding post-oligomerization (25). Although it is unclear whether oligomerization initiates partial stem domain unfolding or vice versa, CD11b has been shown to trigger LukAB oligomerization in solution (28). Given all the CD11b-independent variants have mutations in residues that cluster at the stem/cap interface, we suspected there may be a connection between stem domain unfolding, toxin oligomerization, and the role of CD11b. 2-methyl-2,4-pentanediol (MPD) is a compound that has been used to induce the oligomerization of various *S. aureus* toxins *in vitro* (26, 33), including LukAB (22). By treating LukAB with MPD to induce oligomerization followed by cross-linking with glutaraldehyde, oligomeric complexes can be trapped and subsequently detected in the form of a gel shift by Western blotting. Incubating WT LukAB with 40% MPD results in a shift of the protein to higher molecular weight sizes, presumably corresponding to LukAB oligomers (Fig. 4*A*). However, 20% MPD is not sufficient to induce the formation of WT LukAB oligomers (Fig. 4*A*). In contrast, with the selected alanine variants, we observe that 20% MPD results in the conversion of the majority of LukAB protein to higher molecular weight species (Fig. 4*A*), suggesting that the dimer-oligomer equilibrium is shifted toward oligomers in these variants.

As it is challenging to determine the precise sizes of the high-molecular-weight complexes detected by SDS-PAGE, we next turned to negative stain electron microscopy to visualize the dimer-oligomer equilibrium at the single particle level. We focused on V113A, a variant for which we observed a significant shift in our crosslinking assay. We prepared WT and V113A in Tris-buffered saline (TBS) without MPD, TBS supplemented with 20% MPD and TBS supplemented with 40% MPD, and imaged the samples. Consistent with the crosslinked protein seen by Western blot, we observe oligomers in 20% MPD conditions with V113A, but not for the WT protein under the same conditions (Fig. 4*B*). In the presence of 40% MPD, we observe oligomers for both WT and V113A (Fig. 4*B*), suggesting that both proteins are capable of oligomerizing in MPD, but the barrier to form oligomers is reduced for V113A. 2D class averages show a range of oligomers, including particles that are consistent in shape and size with octamers (Fig. 4, *C*, *E* and *F*). Interestingly, we observed some oligomers of V113A even in the absence of MPD, suggesting that oligomer formation in this variant may occur spontaneously (Fig. 4, *B* and *D*). In the absence of MPD, the V113A oligomers interact non-specifically with each other (Fig. 4, *B* and *D*); the non-specific interactions appear to be relieved in the presence of MPD.

To assess the size of the observed oligomers, we overlaid the crystal structure of the LukAB octameric pore (22) with our 2D classes at the same scale (Fig. 4*G*). The overlay shows that the size of particles in our 2D classes are consistent in size and shape with a LukAB octamer. Taken together, the crosslinking and negative stain EM data show that V113A oligomerizes more readily than WT LukAB.

### Alanine mutations induce a change in LukAB stem domain accessibility

**Figure 5:**
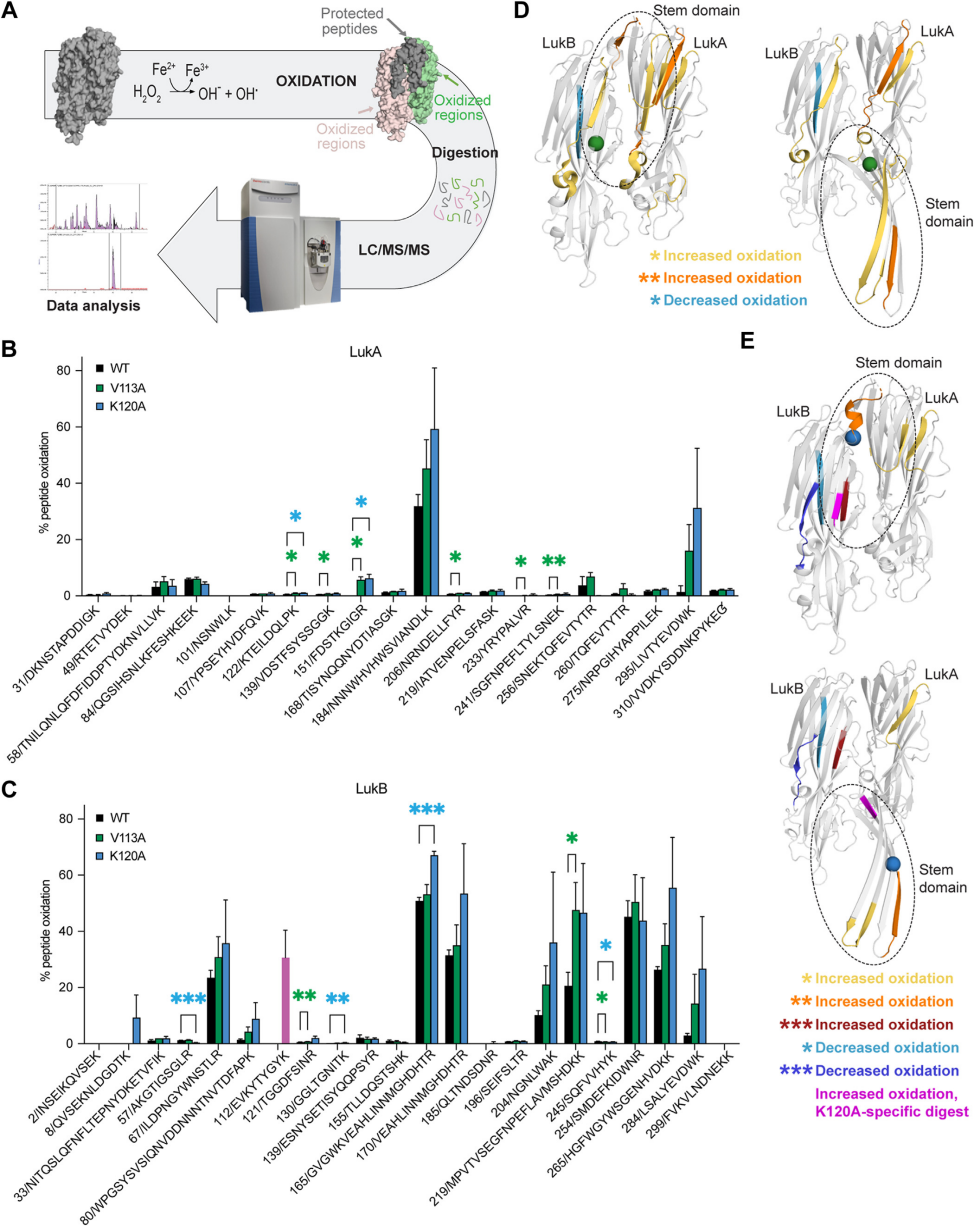
**Oxidative footprinting reveals differentially exposed epitopes.***A*, schematic describing the workflow for LukAB oxidative footprinting. LukAB (*gray* surface model, PDB 5k59 (24)) was incubated with hydrogen peroxide to oxidize surface-exposed residues. The figure is modeling a predicted example; if the stem domain was folded in a stable dimer state, we hypothesize the stem/cap interface will be shielded from solvent (*gray*) while residues exposed to solvent become oxidized (LukA, *green*; LukB, *pink*). LukAB was digested with trypsin, and LC/MS/MS was carried out to determine which peptides are oxidized, probing for differences in solvent accessibility between variants. *B*, bar graph representing the percentage of LukA peptides oxidized for WT, V113A, and K120A. X-axis shows starting residue position followed by single letter amino acid codes. Error bars represent the mean with SD. Statistical significance determined by two-way ANOVA with Dunnett’s multiple comparisons test to WT (∗∗*p* ≤ 0.01; ∗*p* ≤ 0.05; no stars = ns); N = 3 technical replicates. V113A: K122-K131 *p* = 0.0363, V139-K150 *p* = 0.0102, F151-R159 *p* = 0.0168, N206-R215 *p* = 0.0499, Y233-R240 *p* = 0.0470, S241-K255 *p* = 0.0095; K120A: K122-K131 *p* = 0.0239, F151-R159 *p* = 0.0282. Also refer to Extended Data Figure S4. *C*, bar graph representing the percentage of LukB peptides oxidized for WT, V113A and K120A. X-axis shows starting residue position followed by single letter amino acid codes. *Pink* bar represents peptide from K120A that is in a similar region but not digested the same as WT and V113A due to the alanine mutation (See Fig. S8). Error bars represent the mean with SD. Statistical significance determined by two-way ANOVA with Dunnett’s multiple comparisons test to WT (∗∗∗*p* ≤ 0.001; ∗∗*p* ≤ 0.01; ∗*p* ≤ 0.05; no stars = ns); N = 3 technical replicates. V113A: T121-R129 *p* = 0.0025, M219-K240 *p* = 0.0417, S245-K252 *p* = 0.0335; K120A: A57-R66 *p* = 0.0009, G130-K138 *p* = 0.0077, G165-R184 *p* = 0.0003, S245-K252 *p* = 0.0354. Also refer to Extended Data Figure S4. *D*, mapping of statistically significant peptides found in V113A that differ in oxidation levels compared to WT on the LukAB dimer (*left*) and octamer (*right*) for visualization (PDB 5k59 (24), 4tw1 (22)). *Green sphere* shows V113A residue location. *Grey* = no significant difference. *Yellow* represents an increase in peptide oxidation with *p* ≤ 0.05; *Orange* represents an increase in peptide oxidation with *p* ≤ 0.01; *Light blue* represents a decrease in peptide oxidation with *p* ≤ 0.05. Stem domains highlighted with *dashed circle*. *E*, mapping of statistically significant peptides found in K120A that differ in oxidation levels compared to WT on the LukAB dimer (*top*) and octamer (*bottom*) for visualization (PDB 5k59 (24), 4tw1 (22)). *Blue sphere* shows K120 residue location. *Grey* = no significant difference. *Yellow* represents an increase in peptide oxidation with *p* ≤ 0.05; *Orange* represents an increase in peptide oxidation with *p* ≤ 0.01; *Red* represents an increase in peptide oxidation with *p* ≤ 0.001; *Light blue* represents a decrease in peptide oxidation with *p* ≤ 0.05; *Navy blue* represents a decrease in peptide oxidation with *p* ≤ 0.001; *Magenta* represents peptide which has >30% oxidation in K120A, yet this exact peptide is not seen in WT due to digest differences (See Fig. S8). Stem domains highlighted with *dashed circle*.

Considering all top hits from the screen promoting CD11b-independent cytotoxicity concentrate at the stem/cap interface of LukAB, we hypothesized that the alanine mutations may be destabilizing the LukAB stem domain, priming the toxin for oligomerization. To compare stem domain flexibility between WT and the variants, we utilized hydroxyl radical footprinting-mass spectrometry (34, 35) on WT, V113A, and K120A LukAB. Solvent-exposed residues were irreversibly oxidized and detected *via* mass spectrometry (Fig. 5*A*). Differences in the extent of oxidation across samples implied changes in solvent accessibility of that region and were therefore indicative of structural changes.

Sequence coverage was high and consistent for WT, V113A, and K120A proteins, yielding 94.8% and 91.1% coverage for LukA and LukB, respectively. Considering only the peptides used for quantifying the extent of oxidation, we achieved 82.5% and 79.9% coverage for WT LukA and LukB respectively, 81.9% and 78.7% for V113A LukA and LukB, and 72.9% and 78.7% for K120A LukA and LukB. In specific regions, we found significant differences in peptide oxidation levels between WT and the V113A and K120A variants, indicating changes in solvent accessibility in those areas of the protein (Fig. 5, *B* and *C*, Extended data Fig. S4). There were only two peptides that were significantly less oxidized in the variants as compared to WT: Ser245-Lys252 in both variants and Ala57-Arg66 in K120A, both of which are in the cap domain of LukB (Fig. 5, *C*–*E*). The rest of the peptides with significant differences in oxidation levels had increased oxidation in the variants as compared to WT. However, oxidation of V113A and K120A was not higher throughout the entire protein, suggesting the alanine mutation is causing a more localized, not global, change in solvent accessibility.

Taking a closer look at the variants’ peptides that are significantly more oxidized than WT, we find many of these peptides at the stem/cap interface (Fig. 5, *D* and *E*). Lys122-Lys131 LukA, a β strand on the cap side of the stem/cap interface, and Phe151-Arg159 LukA, a peptide that makes up the tip of the stem-loop, were more oxidized in both V113A and K120A, showing these two mutations result in similar changes in those regions (Fig. 5, *B*, *D* and *E*).

We also identified a few peptides that were uniquely more oxidized in V113A LukAB compared to WT. These include Val139-Lys150 LukA, a peptide that makes up half of the LukA stem domain β-strand; Ser241-Lys255 LukA, a β strand at the cap side of the stem/cap interface; Thr121-Arg129 LukB, a peptide that makes up the tip and a quarter of the LukB stem domain; and Met219-Lys240 LukB, a peptide on the cap side of the stem/cap interface (Fig. 5, *B*–*D*). Interestingly, Asn206-Arg215 and Tyr233-Arg240, two peptides within the rim domain loops of LukA, are also more oxidized in V113A (Fig. 5, *B* and *D*). We speculate there are conformational changes in these loops that could be a consequence of stem domain movement.

In the case of K120A, Gly130-Lys138 LukB, which makes up a quarter of the LukB stem domain, was more oxidized than in the WT toxin (Fig. 5, *C* and *E*). Gly165-Lys169 LukB was also more oxidized (Fig. 5, *C* and *E*), and while this peptide is within a β strand on the opposite face of the stem domain, it appears more solvent exposed when the stem domain is unfolded (Fig. 5*E*).

Given that trypsin cleaves protein after lysine and arginine residues, and K120A lost a lysine, the peptides in that region of the protein are cleaved in a different pattern than WT. While we cannot directly compare the oxidation of the peptide encompassing the alanine substitution at position 120 to WT, we see low levels of oxidation overall in the stem domain peptides of WT LukAB, yet Glu112-Lys114 from the K120A variant is very highly oxidized (>30%) (Fig. S8). Altogether, these data demonstrate that these alanine substitutions cause epitopes at the stem/cap interface to be more solvent-accessible than those in WT protein.

## Discussion

CD11b is a critical receptor for LukAB (27) and, along with HVCN1 (Fig. 1*D*) (16), allows LukAB to reach maximum cytotoxic potential and carry out its lytic activity. However, the molecular consequence each receptor has on LukAB and how they contribute to LukAB-mediated pore-formation remains to be elucidated. We performed a comprehensive alanine scanning mutagenesis screen on CD18/HVCN1 CHO to uncover mutations in LukAB that enable enhanced CD11b-independent CC8 LukAB cytotoxicity. The work described herein shows how alanine mutations at the stem/cap interface of LukAB can induce a change in solvent accessibility of peptides specifically localized at that stem/cap interface. One possible reason for increased solvent accessibility is a change in local flexibility. Given that we observe increased solvent accessibility in the stem domain and also an increased propensity for these variants to oligomerize, we propose that a change in flexibility induced by the alanine mutation leads to a premature release of the stem domain, which promotes LukAB to oligomerize more readily, and allows the toxin to maintain full lytic activity in the absence of CD11b.

**Figure 6:**
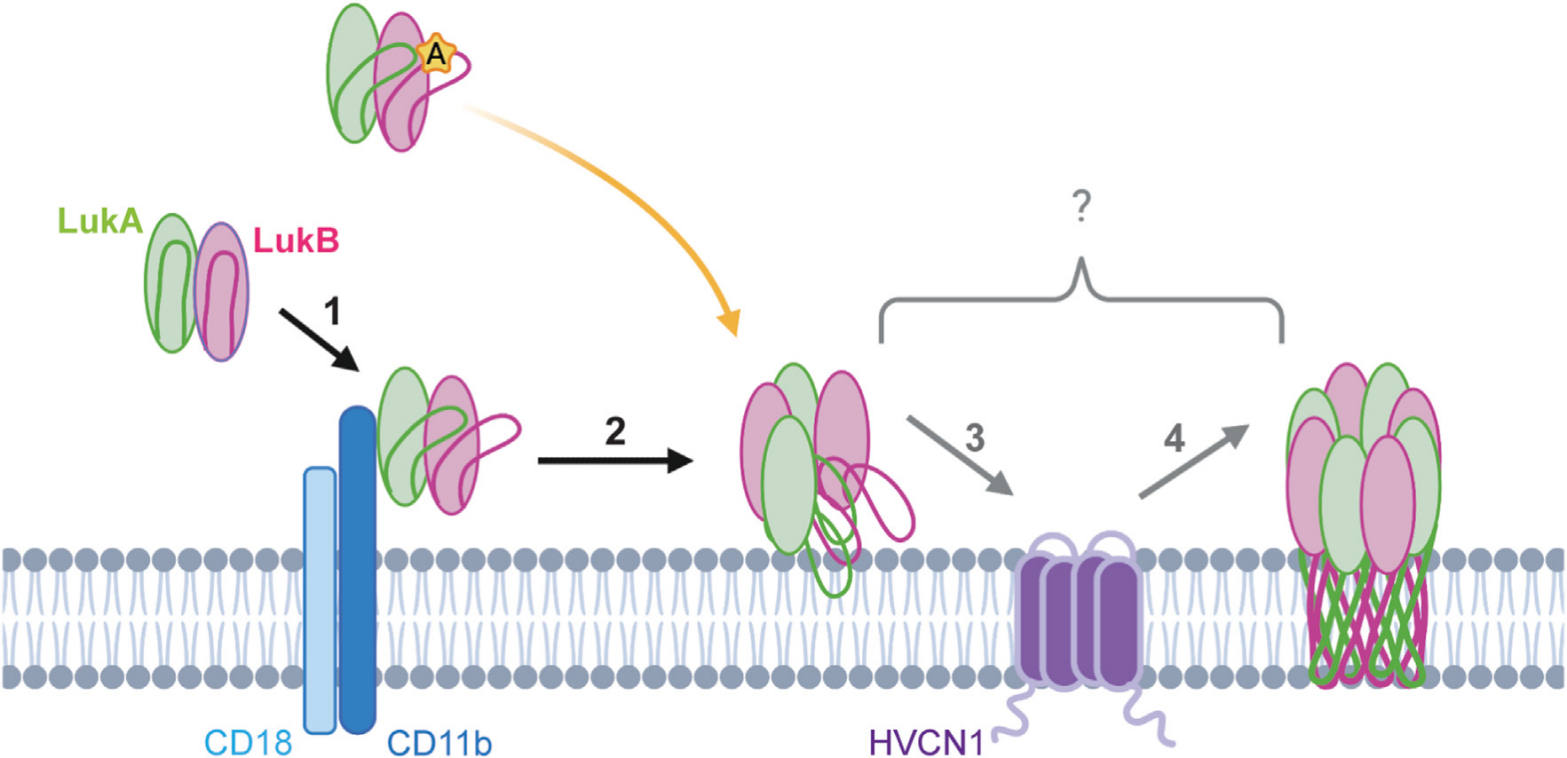
**Proposed model of the CC8 LukAB pore-forming process.** The CC8 LukAB heterodimer is represented as *green* and *pink ovals*, with their corresponding stem domains as lines. Integrin CD18/CD11b is represented as a *light blue* and *dark blue* complex, while proton channel HVCN1 is represented in *purple*. (1) Secreted LukAB dimer engages CD11b, which can trigger a conformational change in the stem domain, allowing it to be more flexible. (2) This flexibility stimulates LukAB to oligomerize into an octamer. Whether LukAB forms a pre-pore or membrane-inserted pore here is unclear, since what stage the stem domains perforate the membrane remains unknown. With an alanine mutation in the stem domain of LukA or LukB (*yellow* star), the LukAB stem domain is more flexible, which primes LukAB to oligomerize and allows for the bypass of CD11b (*yellow arrow*), only requiring HVCN1 to function. (3) HVCN1 is an essential receptor, yet how it contributes to LukAB activity is unknown. (4) Following CD11b and HVCN1 engagement, LukAB forms an octameric β-barrel pore that penetrates the target cell membrane, resulting in cell lysis.

We establish a connection between the stem/cap interface and CD11b receptor dependence, suggesting LukAB binding to CD11b triggers changes in this interface similar to the alanine substitution. We propose the alanine mutation is inducing a change in the local packing at the stem/cap interface, which lowers the activation barrier needed to go from dimer to octamer and eliminates the need for an external catalyst to drive oligomerization. This finding implies a role for CD11b beyond toxin recruitment to the target cell membrane and suggest that CD11b induces the unlatching of the stem domain to promote octameric assembly downstream of binding. We propose this change in stem flexibility is the signal that LukAB has reached its target cell membrane, an upstream cue for the toxin to assemble into an octamer and transition into a pore-forming state (Fig. 6). CD11b I-domain binds at the interface of two adjacent LukAB dimers (28). If we consider the role of CD11b to be facilitating LukAB oligomerization by binding at this interface, then our alanine variants would likely still be unable to function in the absence of CD11b, given there would be no agent to promote assembly. Thus, our mass spectrometry and oligomerization experiments support a model in which conformational changes in the stem domain precede LukAB oligomerization (Fig. 6). However, it is unclear if the oligomers formed by the alanine variants represent a pre-pore or a pore-forming state since it remains unknown when complete stem domain unfolding and membrane insertion takes place in the pore-forming process.

Examination of the non-redundant LukAB protein database failed to find LukAB sequences with alanine at any of the positions identified in our screen (Table S3). We found that the LukAB residues in the stem domain are largely conserved (Table S3). Of the top 30 residues that allow for CD11b-independent cytotoxicity, all are identical in the consensus LukAB sequences for the major *S. aureus* clonal complexes, except for I46 LukA, which is V46 in CC30, CC45, CC75, and CC398, a conservative substitution. This is despite LukAB having a high degree of genetic variability, where allelic variants can have up to 18% differences in amino acids (36). This suggests that LukAB has evolved to preserve the integrity of its stem domain interface and maintain the requirement for two receptors for the majority of LukAB variants, with the exception of CC30 and CC45 LukAB. Perhaps using two receptors allows for more controlled triggering of pore-formation, as our data suggest that our alanine variants oligomerize easier than WT toxin. Moreover, as we observed some spontaneous oligomerization with the alanine variants (Fig. 4, *B* and *D*), it is unknown whether pre-formed oligomers in solution can lyse target cells.

In addition to finding toxins with enhanced activity, our screen also uncovered mutations that are deleterious for LukAB function (Fig. 2*A*, Extended Data Fig. S2). Those toxins that have reduced activity as compared to WT could have lost function because the mutation disrupts oligomerization, hinders the interaction with HVCN1, or prevents pore-formation. All of these possibilities are intriguing and will be exciting to follow up in future studies to further understand the residues critical for LukAB function.

Another outstanding question that remains is what is the role of HVCN1. While we know HVCN1 is sufficient for LukAB to bind cells (Fig. 1*F*), HVCN1 alone does not allow for maximum CC8 LukAB cytotoxicity (Fig. 1*D*). HVCN1 engagement may promote LukAB to undergo a conformational change like CD11b but in a less efficient manner. Other possible roles for HVCN1 include stabilizing the CC8 LukAB pore or facilitating stem domain insertion into the membrane. Further investigation is needed to elucidate this.

In contrast to CC8, CC30 and CC45 LukAB can exhibit high cytotoxic activity without CD11b (16), suggesting HVCN1 can fulfill the role of both receptors with these variants. It will be intriguing to explore the molecular means by which CC30 and CC45 LukAB exploit HVCN1 to drive CD11b-independent cytotoxicity.

In conclusion, the alanine variants described in this study have provided us with new tools to gain considerable insight into how CD11b contributes to the progression of LukAB into its final membrane-embedded state. Further mechanistic understanding of the process of cell targeting and pore-formation by bi-component toxins is needed to identify opportunities to develop novel anti-leukocidin therapeutics and toxoid vaccine candidates.

## Experimental procedures

### Ethics statement

Leukopaks were obtained from de-identified healthy adult donors with informed consent from the New York Blood Center and were used for the isolation of neutrophils. De-identified samples are exempted from the ethics approval requirements by the NYULH Institutional Review Board.

### Bacterial strains

All strains used in this study are listed in Table S4.

### CHO cell lines

CHO-K1 Chinese hamster ovary cells (ATCC CCL-61) stably expressing the CD18 subunit or both CD18/CD11b were kindly provided by Radim Osicka (37). All CHO lines were maintained in Ham’s F12 Nutrient Mixture medium with L-Glutamine (Gibco) supplemented with 10% heat-inactivated Fetal Bovine Serum (FBS), penicillin (100 U/ml), and streptomycin (0.1 mg/ml) (1× P/S) and 300 μg/ml zeocin at 37 °C with 5% CO_2_. The growth media for CD18/CD11b CHO were also supplemented with 600 μg/ml G418. CD18 or CD18/CD11b CHO-K1 lentivirally transduced with HVCN1 (16) were maintained in full medium supplemented with 5 μg/ml puromycin.

### THP-1 cell lines

THP-1 cells were maintained in RPMI 1640 (Corning, 10040CV) supplemented with 10% FBS, penicillin (100 U/ml), and streptomycin (0.1 mg/ml) (1× P/S) and grown at 37 °C with 5% CO_2_. THP-1 shRNA cell lines were generated as described previously (16) and maintained in 10 μg/ml blasticidin. WT THP-1 cells (ATCC TIB-202) were transduced with lentiCRISPRv2 to express non-targeting (HGLibA_64423) or *HVCN1* targeting (HGLibB_22402) sgRNA as described previously (16). Transduced cells were maintained in 1.3 μg/ml puromycin.

### LukAB purification from *S. aureus*

The strain was streaked on Tryptic Soy agar (TSA) + 10 μg/ml Chloramphenicol (Cm). Overnight (O/N) cultures were started in Tryptic Soy broth (TSB) + 10 μg/ml Cm, and subcultured the next day at 1:100 in TSB + 5 μg/ml Cm. Cultures were grown for 5 h at 37 °C/180 rpm, at which point the supernatant was harvested by centrifugation at 6693 rcf/15 min/4 °C. The supernatant was filtered through a 0.2 μm filter and incubated with Ni-Sepharose six Fast Flow resin (Cytiva) for 1 h. The supernatant was passed through a column and washed with 25 mM imidazole in Tris-buffered saline (TBS, 20 mM Tris pH 7.4/150 mM NaCl) followed by a TBS wash and eluted in 500 mM imidazole diluted in TBS. Purified protein was transferred to a Slide-A-Lyzer Dialysis cassette with a 3.5 kD molecular weight cutoff (Thermo Scientific) for dialysis into TBS + 10% glycerol at 4 °C. After O/N dialysis including two buffer exchanges, the protein was filtered through a 0.2 μm filter, protein concentration was measured using a NanoDrop spectrophotometer (Thermo Scientific) and stored at −80 °C.

We also prepared WT CC8 LukAB as an additional protein prep used in Figure 1, *D*–*F*, and Fig. S1 using an AKTA pure chromatography system (Cytiva). Supernatants were harvested as described above. ∼366 mM NaCl was added to the supernatant, and the supernatant was adjusted to pH ∼7.5. The supernatant was loaded onto a HisTrap Excel column (Cytiva) equilibrated in 20 mM Na_2_HPO_4_/500 mM NaCl pH 7.4. The column was washed with 30 ml 20 mM Na_2_HPO_4_/500 mM NaCl/15 mM imidazole pH 7.4, and the protein was eluted in 20 mM Na_2_HPO_4_/500 mM NaCl/500 mM imidazole pH 7.4 using a 25 ml linear gradient collected in 0.5 ml fractions. Protein was dialyzed as described above.

All proteins used throughout the study were purified from *S. aureus*, except the ones used in Figure 2, *A* and *C*, and Fig. S2–S4, which were from *E. coli*.

### Intoxication of THP-1 cells with LukAB variants

THP-1 cells were harvested in RPMI 1640 without phenol red (Gibco, 11835055) + 10% FBS at a concentration of 100,000 cells/90 μl. In all, 10 μl titrated LukAB was incubated with 90 μl (100,000) cells in a tissue culture treated flat bottom 96-well plate at 37 °C/5% CO_2_ for 2 h. PBS or PBS supplemented with 0.2% Triton X-100 (final concentration) were used as controls. After intoxication, 25 μl of supernatant from each well was mixed with 25 μl CytoTox-ONE Homogeneous Membrane Integrity Assay (Promega) in a black-walled clear-bottom 96-well plate in the dark at room temperature (RT) for 15 min, then fluorescence (560 nm excitation, 590 nm emission) was read on an Envision Multilabel Plate Reader (PerkinElmer).

### WT LukAB binding to CHO

Purified CC8 LukAB was titrated in PBS and aliquoted in a tissue culture-treated V-bottom plate. CHO cells were harvested by removing media, washing with PBS, and lifting with 0.05% Trypsin/0.53 mM EDTA in HBSS (Corning). Cells were centrifuged at 390 rcf/5 min/RT to remove trypsin. The pellet was resuspended in Ham’s F12 Nutrient Mixture medium with L-Glutamine (Gibco) supplemented with 10% FBS, penicillin (100 U/ml), and streptomycin (0.1 mg/ml) (1× P/S) and strained through a 40 μm cell strainer. Cell density was determined by Countess II automated cell counter (Thermo Fisher), and cells were diluted to 100,000 cells/90 μl in media. 90 μl (100,000) cells were added to the 10 μl toxin, which was incubated on ice for 10 min to allow the toxin to bind. The plate was centrifuged at 448 rcf/3 min/4 °C, the supernatant was removed, and the cell pellet was resuspended in 150 μl cold PBS. This wash was repeated once more. After the second wash, the cells were resuspended in 25 μl anti-His PE (diluted 1:100 in PBS, Biolegend 362603) and incubated on ice in the dark for 25 min. One hundred fifty microlitre cold PBS was added on top, and the cells were centrifuged again and washed once more. After the last wash, the cells were resuspended in 80 μl FACS fixing buffer (PBS + 2% FBS + 0.05% sodium azide), and samples were run on the Cytoflex Flow cytometer (Beckman Coulter).

Samples were analyzed by FlowJo (version 10.8.1) software (BD). Cells were first gated to remove debris, followed by excluding doublets. Single cells that were PE+ were gated based on PBS-treated cells.

### Cloning cMyc-tagged LukAB

The mature sequence of CC8 LukAB from *S. aureus* Newman was codon optimized for *E. coli* and cloned into a pETDuet-1 vector to include a 6×His tag on the N-terminus of LukA for purification purposes. This vector was provided by Janssen Biotech, Inc.

To add a cMyc tag to the N-terminus of LukB, the pETDuet-1 vector containing 6×His-LukA and LukB was amplified (Phusion, Thermo Scientific) in two halves using the following primer combinations: 5′-gatgaaacgagagaggatgctcacg/5′-atgtatatctccttcttatacttaac and 5′-aaaatcaatagcgaaatcaagcaagt/5′-cgtgagcatcctctctcgtttcatc. DpnI (NEB) was then added to digest the parental plasmid at 37 °C/2 h. Amplified fragments were gel extracted. The following gBlock gttaagtataagaaggagatatacatATGAACTCCGCAGAACAGAAACTGATTAGCGAAGAAGACCTGGGGTCCaaaatcaatagcgaaatcaagcaagt was ordered (Integrated DNA Technologies) to allow for the addition of a cMyc tag with a GS linker, including flanking homology regions to LukAB. The gBlock was assembled with the two vector halves at 50 °C/1 h by Gibson assembly (Gibson Assembly Mix, NEB), followed by transformation into competent *E. coli* DH5⍺ and plating on Lysogeny broth (LB) agar supplemented with 100 μg/ml ampicillin (Amp) for plasmid selection. The clone with the correct sequence was transformed into *E. coli* BL21-DE3(gold).

### Purification of LukAB from *E. coli*

BL21-DE3(gold) *E. coli* expression strain containing LukAB was streaked onto LB agar supplemented with 100 μg/ml Amp for single colonies. O/N cultures were started from 3 to 4 single colonies in 25 ml Terrific Broth (TB) + 100 μg/ml Amp in a 250 ml flask, shaking at 37 °C/180 rpm. The cultures were subcultured the next day 1:20 in 400 ml TB + 100 μg/ml Amp shaking at 37 °C/220 rpm until OD_600_ reached ∼0.6 to 0.8, at which point they were induced with 1 mM IPTG and transferred to 16 °C/220 rpm for O/N expression. Following O/N expression, cultures were harvested by centrifugation at 15,010 rcf/4 °C/15 min, resuspended in 40 ml TBS, transferred to a 50 ml conical, and centrifuged again at 3184 rcf/4 °C/15 min. Pellets were frozen at −80 °C until lysis.

Upon lysis, pellets were resuspended in 18 ml lysis buffer (50 mM Na_2_HPO_4_ pH 7.4, 500 mM NaCl, 10 mM imidazole, 1× Protease inhibitor [Halt Protease Inhibitor Cocktail, EDTA-free, Thermo Scientific], 140 U/μl Benzonase Nuclease [MilliporeSigma], 10% glycerol). Samples were sonicated on ice for 6 × 20 s at 65% amplitude with 30 s breaks between each round of sonication. 2 ml Bugbuster 10× Protein Extraction Reagent (MilliporeSigma) was added to the lysate, and the sample was incubated on ice for 30 min. Cell debris was pelleted at 15,010 rcf/4 °C/30 min, and the supernatant was cleared by filtering through a 0.2 μm filter. The cleared lysate was diluted in 30 ml of 50 mM Na_2_HPO_4_ pH 7.4, 10 mM imidazole, 1× Protease inhibitor (Halt Protease Inhibitor Cocktail, EDTA-free, Thermo Scientific), 10% glycerol, and incubated with ∼2 ml Ni Sepharose six Fast Flow resin (Cytiva) by rocking at 4 °C for 1.5 h. Lysate was loaded onto a column and flow-through was allowed to drip through steadily. The resin was washed with 180 ml 20 mM Na_2_HPO_4_ pH 7.4/75 mM NaCl/25 mM imidazole/10% glycerol, followed by a second wash with 180 ml 20 mM Na_2_HPO_4_ pH 7.4/75 mM NaCl/50 mM imidazole/10% glycerol. Protein was eluted in 30 ml 20 mM Na_2_HPO_4_ pH 7.4/75 mM NaCl/500 mM imidazole/10% glycerol, transferred to a Slide-A-Lyzer Dialysis cassette with a 3.5 kD molecular weight cutoff (Thermo Scientific) for dialysis into TBS + 10% glycerol. After O/N dialysis including three buffer exchanges, the protein was filtered in through a 0.2 μm filter. Protein concentration was measured using a NanoDrop spectrophotometer (Thermo Scientific), and protein was stored at −80 °C.

### Intoxication of PMNs with cMyc-tagged LukAB

LukAB was titrated in PBS, and PMNs were diluted to 200,000 cells/90 μl in RPMI (Gibco) + 10 mM HEPES + 0.5% Human serum albumin (HSA). 90 μl cells (200,000) were added to 10 μl toxin in a tissue culture treated flat bottom 96-well plate, and the plate was incubated at 37 °C/5% CO_2_ for 1 h. PBS and PBS supplemented with 0.2% Triton X-100 (final concentration) were used as controls. 10 μl CellTiter 96 AQueous One Solution (Promega) was added to each well, and incubated at 37 °C/5% CO_2_ for an additional 1 h 30 min. Absorbance at 492 nm was read on an Envision Multilabel Plate Reader (PerkinElmer).

### Synthesis and preparation of LukAB alanine library

The pETDuet-1 co-expression vector which includes a LukA N-terminal 6×His tag and LukB N-terminal cMyc tag described above was used as a template to generate the alanine scanning library (Extended Data Fig. S1). The plasmid encoding WT *lukA* and *lukB* was provided to SynBio Technologies for library synthesis. SynBio generated the full alanine scanning library, where every codon that did not encode an alanine was mutated to encode an alanine. This resulted in 615 plasmids with a single alanine point mutation in either *lukA* or *lukB*. Each plasmid was sequenced to verify the correct mutation. We received the full library of lyophilized plasmids arrayed in a 96-well plate format.

### LukAB alanine screen

Lyophilized plasmids were resuspended in Milli-Q H_2_O to an approximate concentration of 100 ng/μl. 100 ng of plasmid was transformed into *E. coli* BL21(gold)-DE3 competent cells in square well, round bottom 96-well 2 ml deep well blocks (Thermo Fisher, AB0661). After transformation, 1 ml Terrific Broth (TB) + 100 μg/ml Amp for plasmid selection was added to the cells. The blocks shook O/N at 37 °C/220 rpm and were subcultured the next day 1:20 in 1 ml TB + 100 μg/ml Amp. Subcultures were grown at 37 °C/220 rpm until OD_600_ reached ∼0.6 to 0.8, at which point they were centrifuged (3184 rcf/10 min/4 °C) and pellets were resuspended in LB + 10% glycerol for frozen bacterial stocks stored at −80 °C.

For library expression, strains were stamped out on LB agar plates supplemented with 100 μg/ml Amp and incubated at 37 °C O/N. The next day, strains were inoculated in 1 ml TB + 100 μg/ml Amp and grown at 37 °C/220 rpm O/N. The following morning, cultures were subcultured 1:20 in 1 ml TB + 100 μg/ml Amp and grown until OD_600_ reached ∼0.6 to 0.8, at which point they were induced with 1 mM IPTG, and grown for 2 h at 37 °C/220 rpm. After a 2 h expression, cultures were centrifuged at 3184 rcf/10 min/4 °C, and pellets were frozen at −80 °C until lysis.

For lysis, pellets were resuspended in 200 μl lysis buffer (50 mM Na_2_HPO_4_ pH 7.5, 500 mM NaCl, 1× Protease Inhibitor [Halt Protease Inhibitor Cocktail, EDTA-free, Thermo Scientific], 140 U/μl Benzonase Nuclease [MilliporeSigma], 1 mg/ml lysozyme), transferred to a fresh round well, round bottom Nunc 96-well deep well block (Thermo Scientific) and incubated on ice for 1 h. The cells underwent eight freeze/thaw cycles in liquid nitrogen and a 37 °C water bath. Cell debris was pelleted at 3184 rcf/10 min/4 °C, and the supernatant was cleared through a 96-well 0.2 μm filter spin plate (Corning FiltrEX).

### Library screen on CD18/HVCN1+ CHO cells

CHO cells were harvested as previously described. 10 μl *E.* *coli* lysate (5%) containing a different alanine variant was incubated with 90 μl of 100,000 CD18/HVCN1 CHO cells at 37 °C/5% CO_2_ for 1 h in a flat bottom tissue culture treated 96-well plate. CHO cell media and PBS supplemented with 0.2% Triton X-100 (final concentration) were used as controls. Cell lysis was evaluated using CytoTox-ONE Homogeneous Membrane Integrity Assay (Promega) by mixing 25 μl reagent with 25 μl cell supernatant in a black-walled clear-bottom 96-well plate. Plates were incubated in the dark for 15 min, and then fluorescence (560 nm excitation, 590 nm emission) was read on an Envision Multilabel Plate Reader (PerkinElmer).

### ELISA

Plates were coated with 10 μl lysate and 90 μl PBS, and incubated at 4 °C O/N. The next day, plates were washed with 100 μl, 125 μl, and 150 μl 0.05% PBS-T, then blocked with 200 μl 2.5% milk while rocking for 1 h at RT. LukA was detected with an anti-His antibody (Cell Sciences CSI20563C, diluted 1:1000 in 2.5% milk), and LukB was detected with an anti-cMyc antibody (Invitrogen 132500, diluted to 1 μg/ml in 2.5% milk), rocking at RT for 1 h. Plates were then washed 125 μl, 150 μl, and 175 μl 0.05% PBS-T, before rocking in secondary Goat anti-Mouse IgG HRP antibody (Bio-Rad 1706516, diluted 1:4000 in 2.5% milk) at RT for 1 h. Plates were washed again as described before and incubated with 100 μl TMB substrate (Thermo Scientific) in the dark for 10 min. To stop the reaction, 100 μl 2 N Sulfuric acid was added, and absorbance at 450 nm was read on an Envision Multilabel Plate Reader (PerkinElmer).

### Expression and crude purification of top 30 CD11b-independent variants

The top 30 strains that resulted in the highest CD11b-independent lytic activity were streaked out on LB agar + 100 μg/ml Amp and incubated O/N at 37 °C. The next day, O/N cultures were started by inoculating 5 ml LB + 100 μg/ml Amp with 2 to 3 colonies from the plate. Cultures were grown at 37 °C O/N, then subcultured 1:20 in 50 ml TB + 100 μg/ml Amp the next morning, shaking at 37 °C/220 rpm. When OD_600_ reached ∼0.6 to 0.8, cultures were induced with 1 mM IPTG, then grown at 16 °C/220 rpm O/N for expression. The next day, cultures were centrifuged at 3184 rcf/10 min/4 °C, and pellets were frozen at −80 °C until purification.

Frozen cell pellets were resuspended in 5 ml lysis buffer (50 mM Na_2_HPO_4_ pH 7.5, 500 mM NaCl, 10 mM imidazole, 1× Protease Inhibitor [Halt Protease Inhibitor Cocktail, EDTA-free, Thermo Scientific], 140 U/μl Benzonase Nuclease [MilliporeSigma], and 1 mg/ml lysozyme) and underwent eight freeze/thaw cycles in liquid nitrogen and 37 °C water bath. After lysis, Bugbuster 10× Protein Extraction Reagent (MilliporeSigma) was added and rocked at RT for 30 min. Cell debris was pelleted at 3184 rcf/10 min/4 °C, and the supernatant was filtered through a 0.2 μm syringe filter. Cleared lysate was incubated with ∼250 μl Ni Sepharose six Fast Flow resin (Cytiva) diluted in 5 ml 50 mM Na_2_HPO_4_ pH 7.5, 10 mM imidazole, and 10% glycerol at 4 °C for 1 h. The crude purification took place in a 15 ml conical, where tubes were centrifuged at 1791 rcf/4 °C/5 min, and resin was washed once with 15 mM imidazole and twice with 30 mM imidazole diluted in 20 mM Na_2_HPO_4_ pH 7.5/150 mM NaCl. LukAB was eluted from the resin with 0.5 ml 500 mM imidazole diluted in 20 mM Na_2_HPO_4_ pH 7.5/150 mM NaCl. Protein was twice exchanged into TBS + 10% glycerol by dialyzing in a Slide-A-Lyzer MINI Dialysis device with a 3.5 kD molecular weight cutoff (Thermo Scientific) at 4 °C, including O/N dialysis. After dialysis, the protein was filtered through a 0.2 μm Costar Spin-X Centrifuge Tube Filter (Corning), and total protein concentration was measured using a NanoDrop spectrophotometer (Thermo Scientific).

### Cytotoxicity assay of crude purified proteins on CD18/HVCN1+ CHO cells

LukAB was titrated in PBS, and CHO cells were harvested as previously described. 100,000 CD18/HVCN1 CHO cells in 90 μl were added to 10 μl toxin in a flat-bottom tissue culture treated 96-well plate at 37 °C/5% CO_2_ for 1 h. CHO cell media and PBS supplemented with 0.2% Triton X-100 (final concentration) were used as controls. Cell lysis was evaluated using CytoTox-ONE Homogeneous Membrane Integrity Assay (Promega) by mixing 25 μl reagent with 25 μl cell supernatant in a black-walled clear-bottom 96-well plate. The plate was incubated in the dark for 15 min at RT, and then fluorescence (560 nm excitation, 590 nm emission) was read on the Envision Multilabel Plate Reader (PerkinElmer).

### LukAB Western blotting

500 ng crude purified protein was run on a 12% SDS-PAGE. Protein was transferred to a nitrocellulose membrane at 1 A for 1 h. Membranes were blocked in 5% milk diluted in PBS-T (0.05% Tween-20 in PBS) for 1 h. LukA was detected by an anti-His antibody (Cell Sciences CSI20563C, 1:2000), and LukB was detected by an anti-cMyc antibody (Invitrogen 13-2500, 1:4000). After washing the membrane three times with PBS-T, a secondary Alexa Fluor 680 Goat anti-Mouse (Invitrogen A21057, 1:25,000) detected both primary mouse antibodies. After another three washes in PBS-T, the membranes were imaged by LiCor Odyssey DLx.

### Cloning alanine mutants for *S. aureus* expression

LukA or LukB genes with the selected alanine mutation were ordered as gene fragments from Genewiz. To clone a plasmid with an alanine mutation in LukA, both the pOS1 expression vector encoding WT LukAB and the LukA gene fragment containing the alanine mutation were digested with BamHI-HF and XbaI. To clone a plasmid with an alanine mutation in LukB, the pOS1 expression vector encoding WT LukAB and the LukB gene fragment containing the alanine mutation were digested with PstI-HF and XbaI. The digested gene fragment was ligated with the digested pOS1 vector with T4 ligase (NEB), followed by transformation into *E. coli* IM08B competent cells and selection for ampicillin resistance. Clones with the correct sequence were transformed into *S. aureus* Newman ΔΔΔΔ (38).

### Intoxication of CHO, THP-1, and PMNs

LukAB purified from *S. aureus* was titrated in PBS, and 10 μl was aliquoted to a flat bottom, tissue culture treated 96-well plate. Cells were prepared in their respective media: F-12K + 10% FBS + penicillin (100 U/ml), and streptomycin (0.1 mg/ml) (1× P/S) for CHO; RPMI 1640 + 10% FBS + 1× P/S for THP-1; RPMI + 10% FBS for PMNs. 100,000 cells diluted in 90 μl were added to the toxin. Media, PBS, and PBS supplemented with 0.2% Triton X-100 (final concentration) were used as controls. CHO and THP-1 were intoxicated for 1 h at 37 °C/5% CO_2_, after which 25 μl supernatant was added to 25 μl CytoTox-ONE Homogeneous Membrane Integrity Assay (Promega) reagent in a black-walled clear-bottom 96-well plate. The reaction proceeded at RT in the dark for 15 min after which fluorescence was read (560 nm excitation, 590 nm emission). Intoxication of PMNs was allowed for 75 min at 37 °C/5% CO_2_, after which 10 μl CellTiter 96 AQueous One Solution (Promega) was added to each well, and the incubation proceeded for another 75 min at 37 °C/5% CO_2_ before measuring absorbance at 492 nm. All plates were read on an Envision Multilabel plate reader (PerkinElmer).

### Generation of Alexa Fluor 488 labeled LukAB

LukAB with select alanine mutations were cloned to include an N-terminal Cysteine (21) to allow for Alexa Fluor 488 (AF488) conjugation. Cysteine was added to the N-terminus following the signal sequence and 6×His-tag by amplifying LukAB from a pOS1 expression plasmid with the forward primer 5′-CCCCGGATCCTGTAATTCAGCTCATAAAGACTCTCAAG-3′ and universal M13 reverse. Amplified sequences and the pOS1 vector were digested with BamHI-HF and PstI-HF, ligated with T4 ligase (NEB) and transformed into *E. coli* IM08B selected by ampicillin resistance. Clones with the correct sequence were transformed into *S. aureus* Newman ΔΔΔΔ (38). Protein was purified from *S. aureus* as previously described.

Purified protein was incubated with 5-fold molar excess Alexa Fluor 488 C_5_ maleimide (Invitrogen A10254) with rocking at RT for 2 h. The reaction was quenched by adding 10-fold molar excess dithiothreitol (DTT). To remove excess dye, the labeled protein was transferred to a Slide-A-Lyzer Dialysis Casette with a 20-kD molecular weight cutoff (Thermo Scientific) for dialysis in TBS + 10% glycerol at 4 °C for three buffer exchanges, including O/N dialysis. After dialysis, protein was filtered through a 0.2 μm Costar Spin-X Centrifuge Tube Filter (Corning), and protein concentration was measured using a NanoDrop spectrophotometer (Thermo Scientific). 500 ng protein was run on a 12% SDS-PAGE, and AF448 labeling and protein normalization were confirmed by AF488 blot and InstantBlue Coomassie protein stain (Abcam), respectively (Fig. S7*A*). The activity of the labeled toxins was confirmed by cytotoxicity assays (Fig. S7*B*) on PMNs as previously described.

### AF488 LukAB binding to CHO

Purified AF488 labeled LukAB was titrated in PBS and aliquoted in a tissue culture–treated V-bottom 96-well plate. CHO cells were harvested as previously described. Cells were diluted to 100,000 cells/90 μl in media + 2.3% Bovine serum albumin (BSA, Fisher BioReagents). 90 μl (100,000) cells were added to 10 μl toxin (final 2% BSA), which was incubated on ice for 5 min to allow the toxin to bind. The plate was centrifuged at 448 rcf/3 min/4 °C, supernatants were removed, and the cell pellets were resuspended in 150 μl cold PBS. This wash was repeated once more. After the second wash, the cells were resuspended in 80 μl FACS fixing buffer (PBS + 2% FBS + 0.05% sodium azide) and samples were run on the Cytoflex Flow cytometer (Beckman Coulter).

Samples were analyzed by FlowJo (version 10.8.1) software (BD). Cells were first gated to remove debris, followed by excluding doublets. Single cells that were AF488+ were gated based on PBS-treated cells.

### HVCN1 pull-down

Strep-tagged HVCN1 was expressed and purified from *E. coli* and exchanged into amphipol (A8-35, Anatrace) as previously described (16). LukS and LukF were purified from *S. aureus* as previously described (39). For the pull-down, ∼400 μl Streptactin XT four Flow High Capacity resin (IBA Life Sciences) was first equilibrated in 20 mM Tris pH 8/150 mM NaCl. Half the resin was incubated with 240 μg purified HVCN1 for 1 h at 4 °C with rocking, while the other half was incubated with TBS (20 mM Tris pH 8/150 mM NaCl) for an empty resin control. After allowing HVCN1 to bind the resin, the resin (HVCN1-bound or empty) was equally distributed to eight wells in a 96-well V-bottom tissue-culture treated plate. The plate was spun at 3738 rcf/2 min/4 °C, and the supernatant was removed. The plate was then washed with 150 μl TBS + 1 mM EDTA and spun again to remove excess HVCN1. 20 μg toxin (LukAB or LukSF) in 200 μl (or 200 μl TBS) was added to the resin (HVCN1-bound or empty) and allowed to bind on ice for 5 min. After a 5-min incubation, the plate was spun at 3738 rcf/2 min/4 °C, and the supernatant was removed. The plate was washed with 200 μl TBS + 1 mM EDTA and spun again. This wash was repeated three times to remove excess toxins. After the fourth wash, the resin was resuspended in 100 μl TBS + 1 mM EDTA + 50 mM biotin to elute HVCN1 from the resin. Each sample was transferred to a 0.2 μm spin filter to separate the protein from the resin. The elution was resuspended in 5× SDS loading dye, and 15 μl was loaded on a 4 to 20% SDS-PAGE. The gel was stained with InstantBlue Coomassie protein stain (Abcam) to visualize the protein. We evaluated binding by comparing the relative intensities of toxin to HVCN1 between WT LukAB and the alanine variants.

### MPD incubation, glutaraldehyde crosslinking, and Western blot

Purified LukAB was dialyzed into 20 mM HEPES pH 7.5/150 mM NaCl O/N at 4 °C to remove Tris. 2 μg protein was incubated with buffer control (20 mM HEPES pH 7.5/150 mM NaCl), buffer + 20% MPD or buffer + 40% MPD at RT for 30 min. After MPD incubation, samples were crosslinked with 0.01% glutaraldehyde (or received buffer control) at 37 °C for 15 min. The reaction was stopped with 0.1 M Tris pH 8. Samples were diluted in SDS dye, boiled, and run on a 4 to 20% SDS-PAGE (BioRad). Protein was transferred to a nitrocellulose membrane at 1 A for 1 h. Membranes were blocked in 5% milk diluted in PBS-T (0.05% Tween-20 in PBS) for 1 h, followed by three washes in PBS-T. The His-tag on LukA was detected by Western blot using an anti-His antibody (Cell Sciences CSI20563C, 1:2000). After washing the membrane with PBS-T three times, a secondary Goat anti-Mouse Alexa Fluor 680 detected the primary antibody (Invitrogen A21057, 1:25,000). After another three washes in PBS-T, membranes were imaged by LiCor Odyssey DLx.

### Negative stain electron microscopy

Freshly purified protein was dialyzed into TBS. Protein was diluted to 0.03 mg/ml in TBS without MPD (control), TBS + 20% v/v MPD, or TBS + 40% v/v MPD at RT for 45 min. Three microlitre protein was applied to a carbon-coated grid that was freshly glow discharged for 30 s. Excess protein was blotted off, and 3 μl 2% uranyl formate stain was applied to the grid and blotted off. This stain application was repeated four times. Grids were imaged on a Talos L120C transmission electron microscope (FEI). Data were collected using Digital Micrograph at a magnification of 73,000× corresponding to a pixel size of 2 Å/pixel, and 51 micrographs for each dataset were processed using Relion 3.1.0 (40); CTF estimation was not performed. A subset of particles were picked manually. 2D classification of the manually picked particles resulted in classes that were subsequently used for template-based picking. Particles from all micrographs were picked using template-based picking with standard default options (a picking threshold of 0.05, a maximum standard deviation threshold on noise of 1.1, and filtering the references to 20 Å). After template-based picking on the entire dataset, particles were extracted using a box size of 140 pixels, and 2D classification was performed (see Extended Data Fig. S3) with default parameters using a mask size of 260 Å and a regularization parameter of T = 2. The resulting 2D classes were visually inspected to curate particles, removing obvious junk particles. After iterative 2D classification, a final stack of particles was used for a final round of 2D classification. Two replicates were performed for each sample from two independent protein purifications.

### Overlay of the LukAB octamer with negative stain 2D classes

To assess the size of the oligomer, we used ChimeraX (41) to overlay the CC8 LukAB octamer (22) (PDB 4tw1) and the 2D classes. The PDB was manually moved such that the PDB intersected with the 2D plane of the class, and translated and rotated manually to overlay with the density observed in the 2D class.

### Hydroxyl radical footprinting mass spectrometry

Three different buffers are needed for the reaction: standard buffer, standard-EDTA buffer, and quench solution. The standard buffer consisted of 25 mM HEPES and 50 mM NaCl at pH 8. Standard-EDTA buffer consisted of 25 mM HEPES, 50 mM NaCl, and 330 mM EDTA at pH 8. Quench solution was prepared with final concentrations of 0.1 ng/μl catalase, 68 μM thiourea, and 34 μM methioninamide (34). The standard and standard-EDTA buffers can be prepared in advance and stored at RT.

The hydrogen peroxide and Fe(II)-EDTA reagents were prepared fresh at the time of the reaction. A 600 mM hydrogen peroxide stock solution was prepared in standard buffer. A 300 mM Fe(II)-EDTA stock solution was prepared in standard-EDTA buffer using ammonium iron (II) sulfate hexahydrate. The stock solutions were then serially diluted so three different reagent concentrations were achieved: 600, 300, 150 mM hydrogen peroxide and 300, 150, and 75 mM Fe(II)-EDTA. A clear 96-well plate was used to facilitate the dilutions and the transfer of reagents for the reaction. Column 1 rows A-C contained the hydrogen peroxide dilutions in order of highest to lowest concentration. Column 2 rows A-C contained the Fe(II)-EDTA dilutions in order of highest to lowest concentration. Row D of columns 1 and 2 contained blank standard and standard-EDTA buffer, respectively. This dilution plate made it easier to produce hydroxyl radicals from hydrogen peroxide by Fe(II)-EDTA mediated Fenton chemistry as a function of oxidizing reagents concentration.

In all, 155 to 260 μg of each protein sample was diluted to 600 μl with 20 mM Tris pH 7.4. The sample plate was prepared by aliquoting 45 μl of each protein sample into three columns and four rows of a black 96-well plate (12 wells total per protein sample).

To perform the oxidative footprinting reaction, the sample plate was mounted on a mixer, and 2.5 μl of the hydrogen peroxide dilutions (column 1 of the dilution plate) followed by 2.5 μl of the Fe(II)-EDTA dilutions (column 2 of the dilution plate) were added to column 1 of the sample plate. The plate was incubated on the mixer for 2 min at RT and ∼600 rpm. The reaction was then quenched with 50 μl of quench solution. This reaction allowed for one replicate of each dose of reagent and one control sample. These 2-min reactions were repeated for three replicates of each mutant. Since the Fe(II)-EDTA only has a 30-min lifetime, this solution was re-prepped before the expiration time.

To remove excess reagents from the sample, each sample was cleaned using a C4 spin column (Harvard Apparatus). All samples were acidified to pH 2 with 10% trifluoroacetic acid (TFA). Spin columns were prepared with methanol and acetonitrile (ACN), followed by three washes with 0.1% TFA. Samples were completely loaded onto respective spin columns and rinsed 3× with 0.1% TFA. Proteins were then eluted with 40% ACN/0.5% acetic acid and 80% ACN/0.5% acetic acid (34). All cleaned samples were dried down to remove organic solvents.

All samples were reconstituted in 100 mM ammonium bicarbonate at pH 8. Samples were reduced with 2 μl 0.2 M dithiothreitol for 1 h at 57 °C, then alkylated with 2 μl 0.5 M iodoacetamide for 45 min in the dark at RT (34). All samples were digested with 250 ng of trypsin O/N at RT. To quench the digestion, samples were acidified to pH 2 with 10% TFA.

Samples were analyzed using an Evosep in line with a Thermo Scientific QE-HFX mass spectrometer. Evosep tips were prepared with an ACN/0.1% formic acid rinse, isopropanol incubation, and 0.1% formic acid rinse. One percent of the sample was loaded onto prepared Evosep tips with 30 fmol PRTC (Pierce Peptide Retention Time Calibration mixture). Tips were rinsed two times with 0.1% formic acid before loading onto Evosep for analysis. Peptides from each sample were eluted using the 15SPD (88 min) Evosep method (Solvent A was 0.1% Formic acid, Solvent B was ACN/0.1% Formic acid). Eluted peptides were analyzed *via* a top20 MS/MS method. High-resolution MS1 scans were acquired with a scan range of 400 to 1500 m/z, 60 K resolution, 1e6 AGC target, and 50 ms injection time. The top 20 precursors were fragmented by HCD with an NCE of 27 and analyzed with a 45 k resolution MS2 scan (2e5 AGC target and 100 ms injection time). Dynamic exclusion was enabled and after one scan the precursor was excluded for 5 s.

All MS/MS spectra were searched against the Uniprot *S. aureus* database using Sequest within Proteome Discoverer 1.4 (Thermo Fisher) to determine the overall purity of the sample. MS/MS spectra were searched again using the oxidative footprinting node of Byos (Protein Metrics) against the respective LukA/B proteins in each sample. Spectra were searched fully tryptic allowing for two missed cleavages, maximum precursor mass of 8000 Da, 10 ppm precursor tolerance, 15 ppm fragment ion tolerance, 1% FDR, manual score cut of 300, two common modification maximum, and one rare modification maximum. Trioxidation at cysteine, dioxidation at cysteine/methionine/tryptophan, and oxidation at isoleucine/lysine/leucine/glutamine/valine were set as common1 modifications. Oxidation at methionine/cysteine/aspartic acid/phenylalanine/histidine/phenylalanine/tryptophan/tyrosine was set as a common2 modification. pyroGlu at N-term glutamine and N-term glutamic acid was set as a rare1 modification. All peptides were manually validated and integrated using the Byos platform. Graphs were generated by determining the percent total oxidation for each peptide in each sample and subtracting the percent oxidation in the control samples. Each dose was conducted in triplicate (Refer to Extended Data Fig. S4). Data in Figure 5 represents 300 mM hydrogen peroxide treatment, with the average and standard deviation presented.

### Residue conservation analysis

To obtain all publicly available LukA and LukB sequences, BLAST (Basic Local Alignment Search Tool) blastp (42) v2.13.0 was used with an e-value threshold of 10^−8^ to search for LukA and LukB protein sequences within the non-redundant protein sequences databases (nr), filtering results to only include *S. aureus* proteins from the RefSeq and INDSC databases. The results of the blastp searches were further filtered to obtain high-confidence hits using a 90% threshold for both percent identity and percent query coverage per high-scoring segment pair. To obtain residue counts for all distinct protein chains in the dataset, a multiple sequence alignment was computed with MAFFT v4.471 (43) and residue counts were tabulated for this subset. We identified 305 distinct LukA protein variants and 280 distinct LukB variants.

### Statistical analysis

GraphPad Prism nine was used for all statistical analyses.

## Data availability

All data are contained within the manuscript. The mass spectrometric raw files are accessible at https://massive.ucsd.edu under accession MassIVE MSV000092897 and at www.proteomexchange.org under accession PXD045499.

## Supporting information

This article contains supporting information (16, 39).

## Conflict of interest

V. J. T. is an inventor on patents and patent applications filed by New York University, which are currently under commercial license to Janssen Biotech Inc. Janssen Biotech provides research funding and other payments associated with the licensing agreement. All other authors declare no conflict of interest.

## Supporting information

Supporting Information

Extended data Figure 2

Extended data Figure 3

Extended data Figure 4
