# Supplementary material for: Unlatching of the stem domains in the Staphylococcus aureus pore-forming leukocidin LukAB influences toxin oligomerization
Source: J Biol Chem. 2023 Oct 4;299(12):105321. doi: 10.1016/j.jbc.2023.105321 (PMC10665946; doi:10.1016/j.jbc.2023.105321)
Supplement: Supporting Information [file mmc1.docx]

**SUPPORTING INFORMATION**

**Supporting Figures**

**
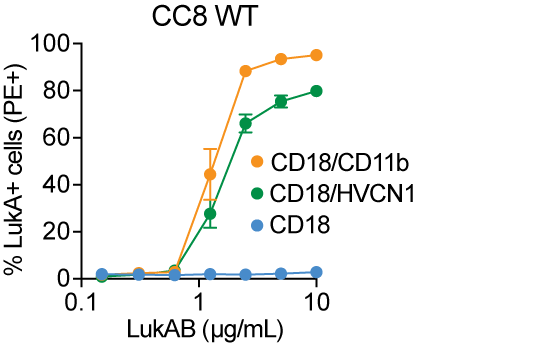
**

**Figure S1: LukAB binds both CD11b and HVCN1 independently of the other receptor**

Binding of purified WT CC8 LukAB to CHO cells stably expressing CD18, CD18/CD11b or CD18/HVCN1. LukA was detected using an anti-His PE antibody, and binding was measured by percent LukAB positive (PE+) cells. Error bar represent SEM. Data is representative of 6 independent experiments using 2 different protein preps for an N = 12.


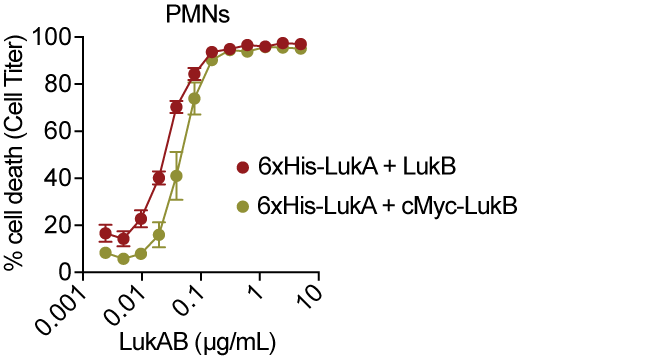


**Figure S2: His and cMyc tagged LukAB is active**

Intoxication of primary human PMNs with purified CC8 6xHis-LukA with or without a LukB N-terminal c-Myc tag. Error bars represent SEM; N = 3. LC50 of LukAB with untagged LukB = 0.028 μg/mL, LC50 of LukAB with cMyc tagged LukB = 0.047 μg/mL.


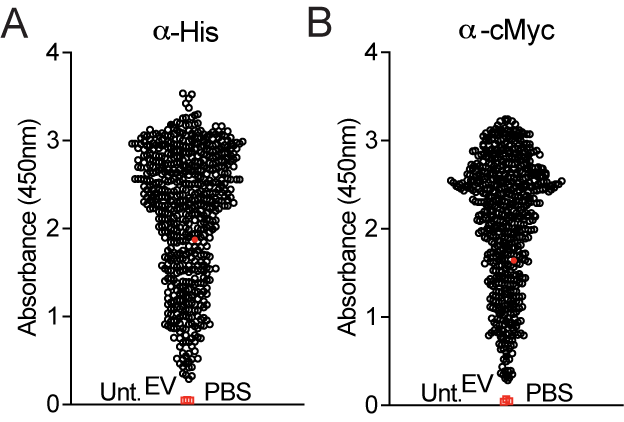


**Figure S3: Production of LukA and LukB in cell lysates**

1. ELISA measuring production of LukA in the cleared cell lysate using an anti-His antibody. Average production of WT LukA from 8 independent expressions marked in filled red circle. Each black dot represents the production of LukA from a different alanine variant resulting from one expression. Untransformed cells (Unt.), Empty vector (EV), and PBS controls marked in outlined red squares.
2. ELISA measuring production of LukB in the cleared cell lysate using an anti-cMyc antibody. Average production of WT LukB from 8 independent expressions marked in filled red circle. Each black dot represents the production of LukB from a different alanine variant resulting from one expression. Untransformed cells (Unt.), Empty vector (EV), and PBS controls marked in outlined red squares.


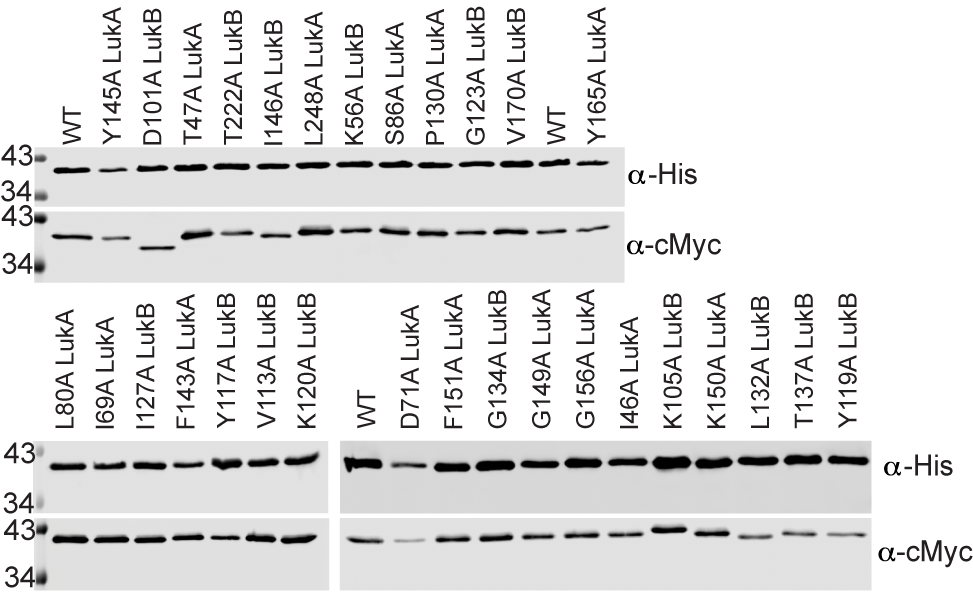


**Figure S4: LukA and LukB production from crude purification**

Western blots confirming LukA and LukB presence in batch purifications. LukA detected with anti-His antibody, LukB detected with anti-cMyc antibody. 500ng total protein loaded.


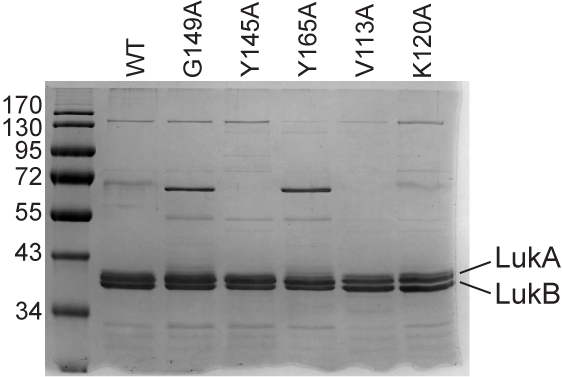


**Figure S5: Purified LukAB WT and variants**

Representative gel depicting LukAB WT and select variants purified from *S. aureus*. 750 ng protein loaded, Coomassie stained.


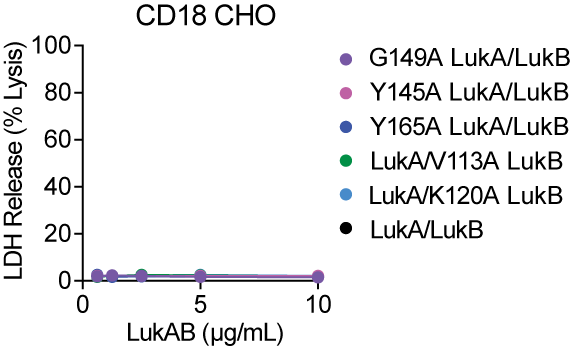


**Figure S6: CHO transduced with CD18 are not susceptible to LukAB**

Cytotoxicity of purified WT and select LukAB variants on CD18 CHO cells. Cell death measured by LDH release. Error bars are present but very small, representing SEM; N = 4.
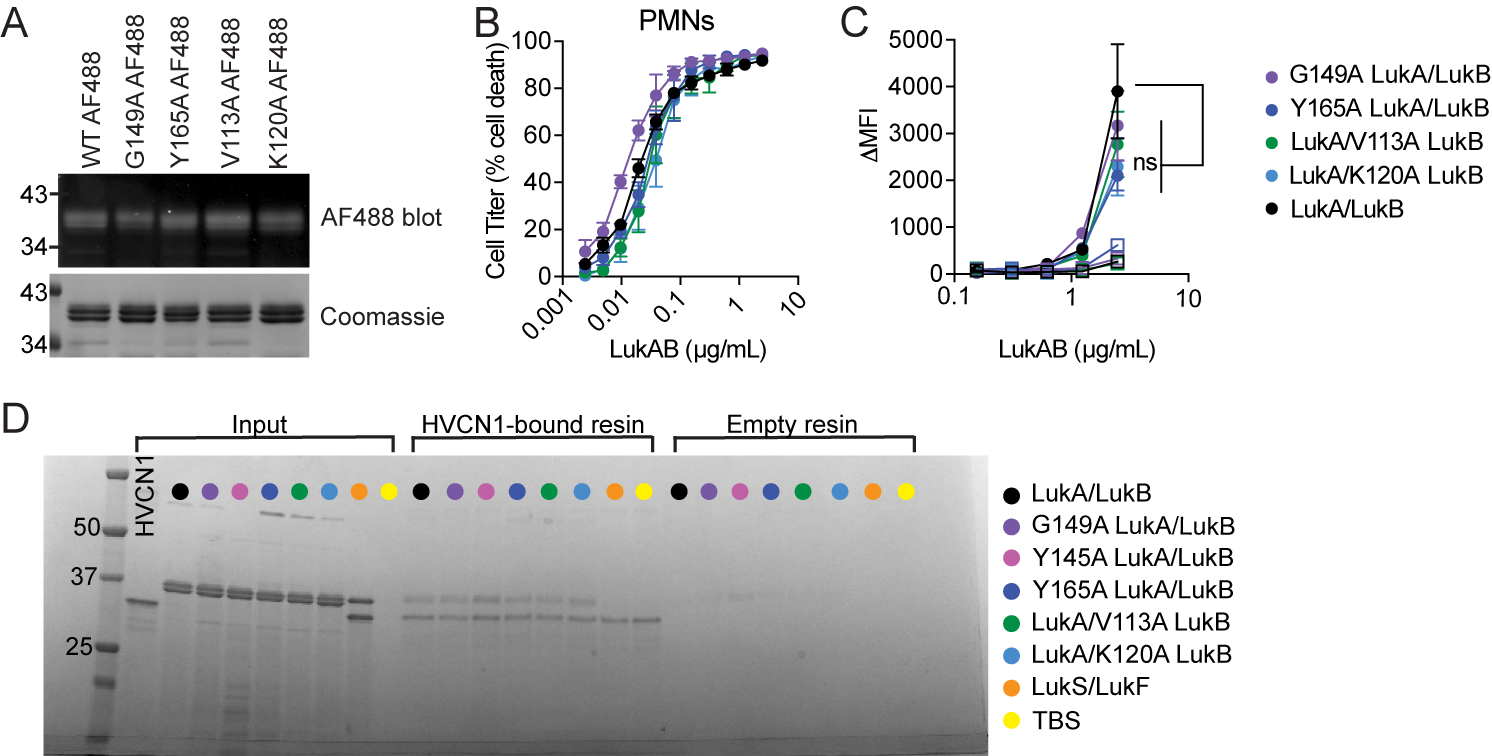


**Figure S7: LukAB alanine variants to not have enhanced binding to HVCN1**

1. AF488 blot (top) and Coomassie stain (bottom) of 500 ng purified AF488 labeled LukAB used in binding assay.
2. Intoxication of primary human PMNs with AF488 labeled LukAB demonstrates the toxins maintain cytotoxic activity with Alexa Fluor labeling. Cell death measured by CellTiter. Error bars represent SEM; N = 5 independent donors.
3. Binding of AF488 labeled WT and select LukAB alanine variants to CD18/HVCN1 CHO (filled circles) or control CD18 CHO (empty squares). Binding measured by ΔMFI with PBS signal subtracted. Statistical significance determined by two-way ANOVA with Dunnett’s multiple comparisons test of each variant compared to WT at the highest concentration in the CD18/HVCN1 CHO binding samples (ns = not significant). Error bars represent SEM; N = 5.
4. Pull-down of WT LukAB and alanine variants with HVCN1. LukSF and TBS (no toxin) were included as controls. Input samples show purified protein used in the assay. HVCN1-bound resin samples represent the elution from the resin after toxin (or TBS) was allowed to bind and excess protein was washed away. Empty resin (no HVCN1 bound) was included to assess nonspecific binding of the toxins to the resin. We observe minimal nonspecific binding of Y145A LukAB to the empty resin, which could result in an overestimation of Y145A LukAB in the HVCN1-bound resin sample. 750 ng input protein loaded, 12% of pull-down elution loaded, Coomassie stained. There was variability in resin loss during the assay, thus protein load was not normalized. Gel is representative of N = 4 independent experiments.


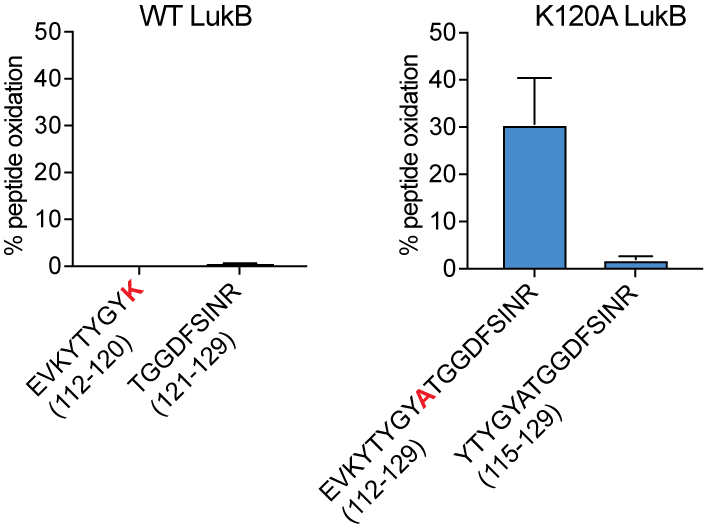


**Figure S8: Peptide digests surrounding K120**

A closer look at the peptides surrounding K120 in WT CC8 LukAB and K120A in the variant. K120 or A120 residues highlighted in red. Error bars represent SD from N = 3 technical replicates.

**Supporting Tables**

**Table S1: LC50 of select CC8 LukAB variants on CD18/HVCN1 CHO**

| **Variant** | **LC50 (μg/mL)^1^** |
| --- | --- |
| G149A LukA | 1.22 (±0.14) |
| Y145A LukA | 1.22 (±0.12) |
| Y165A LukA | 0.80 (±0.06) |
| V113A LukB | 1.76 (±0.07) |
| K120A LukB | 1.24 (±0.13) |

^1^Data from 4 independent experiments. Values represent mean LC50 ± standard deviation from replicates.

**Table S2: LC50 values of WT and select CC8 LukAB variants on primary human PMNs**

| **Variant** | **LC50 (μg/mL)** ^1^ |
| --- | --- |
| WT | 0.09 (±0.09) |
| G149A LukA | 0.09 (±0.09) |
| Y145A LukA | 0.29 (±0.10) |
| Y165A LukA | 0.18 (±0.11) |
| V113A LukB | 0.07 (±0.05) |
| K120A LukB | 0.01 (±0.005) |

^1^Data from 11 independent donors. Values represent mean LC50 ± standard deviation from replicates.

**Table S3. Conservation analysis of deposited LukAB sequences**

| **Residue of Interest^1^** | **Number of occurrences^2^** | **Alternate residue; occurrence^3^** |
| --- | --- | --- |
| I46 LukA | 279 | F; 1 V;25 |
| T47 LukA | 304 | I;1 |
| I69 LukA | 305 |  |
| D71 LukA | 305 |  |
| L80 LukA | 305 |  |
| S86 LukA | 303 | I;1 P;1 |
| P130 LukA | 304 | S;1 |
| F143 LukA | 304 | Deletion;1 |
| Y145 LukA | 304 | Deletion;1 |
| G149 LukA | 304 | Deletion;1 |
| K150 LukA | 304 | Deletion;1 |
| F151 LukA | 304 | Deletion;1 |
| G156 LukA | 302 | D;1 V;1 Deletion;1 |
| Y165 LukA | 303 | H;1 X;1 |
| L248 LukA | 305 |  |
| K56 LukB | 280 |  |
| D101 LukB | 276 | G;1 N;3 |
| K105 LukB | 279 | E;1 |
| V113 LukB | 280 |  |
| Y117 LukB | 279 | H;1 |
| Y119 LukB | 280 |  |
| K120 LukB | 280 |  |
| G123 LukB | 280 |  |
| I127 LukB | 278 | M;1 V;1 |
| L132 LukB | 279 | F;1 |
| G134 LukB | 279 | R;1 |
| T137 LukB | 279 | I;2 |
| I146 LukB | 280 |  |
| V170 LukB | 279 | I;1 |
| T222 LukB | 279 | N;1 |

All LukAB sequences were condensed to 305 unique LukA sequences and 280 unique LukB sequences.

^1^Column 1 lists the top 30 residue hits from the alanine screen.

**^2^**Column 2 lists the number of times that residue is the consensus amino acid in all unique LukA or LukB sequences.

**^3^**Column 3 lists other residues, if any, that are found at that position and the number of unique sequences it is found. X represents an unknown amino acid, where the translated nucleotide sequence was AGN.

**Table S4. Bacterial strains used in this study**

| **Strain** | **Source** | **Identifier** |
| --- | --- | --- |
| *S. aureus* Newman ΔΔΔΔ pOS1_PlukAB-lukAs.s.-6xHis-LukAB CC398 | (16) | VJT 46.54 |
| *S. aureus* Newman ΔΔΔΔ pOS1_PlukAB-lukAs.s.-6xHis-LukAB CC75 | (16) | VJT 47.16 |
| *S. aureus* Newman ΔΔΔΔ pOS1_PlukAB-lukAs.s.-6xHis-LukAB CC45 | (16) | VJT 46.33 |
| *S. aureus* Newman ΔΔΔΔ pOS1_PlukAB-lukAs.s.-6xHis-LukAB CC30 | (16) | VJT 46.19 |
| *S. aureus* Newman ΔΔΔΔ pOS1_PlukAB-lukAs.s.-6xHis-LukAB CC8 | (16) | VJT 46.28 |
| *S. aureus* Newman ΔΔΔΔ pOS1_PlukAB-lukAs.s.-6xHis-LukAB CC5 | (16) | VJT 46.35 |
| *S. aureus* Newman ΔΔΔΔ pOS1_PlukAB-lukAs.s.-6xHis-LukAB CC1 | (16) | VJT 46.29 |
| *E. coli* BL21-DE3(gold) pETDuet-1 6xHis-LukA-LukB CC8 | This study | VJT 62.64 |
| *E. coli* BL21-DE3(gold) pETDuet-1 6xHis-LukA-cMyc-LukB CC8 | This study | VJT 66.95 |
| *E. coli* DH5α pETDuet-1 Empty Vector | Novagen | VJT 21.91 |
| *E. coli* BL21-DE3(gold) pETDuet-1 6xHis-LukA-cMyc-LukB CC8 Alanine Library | This study | Extended Data Fig 2 |
| *S. aureus* Newman ΔΔΔΔ pOS1_PlukAB-lukAs.s.-6xHis-G149A LukA-LukB CC8 | This study | VJT 84.78 |
| *S. aureus* Newman ΔΔΔΔ pOS1_PlukAB-lukAs.s.-6xHis-Y145A LukA-LukB CC8 | This study | VJT 84.82 |
| *S. aureus* Newman ΔΔΔΔ pOS1_PlukAB-lukAs.s.-6xHis-Y165A LukA-LukB CC8 | This study | VJT 84.84 |
| *S. aureus* Newman ΔΔΔΔ pOS1_PlukAB-lukAs.s.-6xHis-LukA-V113A LukA CC8 | This study | VJT 85.56 |
| *S. aureus* Newman ΔΔΔΔ pOS1_PlukAB-lukAs.s.-6xHis- LukA-K120A LukB CC8 | This study | VJT 85.58 |
| *S. aureus* Newman ΔΔΔΔ pOS1_PlukAB-lukAs.s.-6xHis-NtermCysteine- LukA-LukB CC8 | This study | VJT 86.31 |
| *S. aureus* Newman ΔΔΔΔ pOS1_PlukAB-lukAs.s.-6xHis-NtermCysteine-G149A LukA-LukB CC8 | This study | VJT 86.22 |
| *S. aureus* Newman ΔΔΔΔ pOS1_PlukAB-lukAs.s.-6xHis-NtermCysteine-Y165A LukA-LukB CC8 | This study | VJT 86.24 |
| *S. aureus* Newman ΔΔΔΔ pOS1_PlukAB-lukAs.s.-6xHis-NtermCysteine- LukA-V113A LukB CC8 | This study | VJT 86.27 |
| *S. aureus* Newman ΔΔΔΔ pOS1_PlukAB-lukAs.s.-6xHis-NtermCysteine- LukA-K120A LukB CC8 | This study | VJT 86.28 |
| *S. aureus* Newman ΔΔΔΔ pOS1_PlukAB-lukAs.s.-6xHis-LukS | (39) | VJT 37.05 |
| *S. aureus* Newman ΔΔΔΔ pOS1_PlukAB-lukAs.s.-6xHis-LukF | (39) | VJT 37.34 |
| *E. coli* C43 pET24a+_HVCN1_SAlinker_Strep | (16) | VJT 73.21 |

**Extended Data Figure 1: Amino acid sequence of CC8 6xHis-LukA/cMyc-LukB**

**LukA:**

MNSA**HHHHHHGS**HKDSQDQNKKEHVDKSQQKDKRNVTNKDKNSTAPDDIGKNGKITKRTETVYDEKTNILQNLQFDFIDDPTYDKNVLLVKKQGSIHSNLKFESHKEEKNSNWLKYPSEYHVDFQVKRNRKTEILDQLPKNKISTAKVDSTFSYSSGGKFDSTKGIGRTSSNSYSKTISYNQQNYDTIASGKNNNWHVHWSVIANDLKYGGEVKNRNDELLFYRNTRIATVENPELSFASKYRYPALVRSGFNPEFLTYLSNEKSNEKTQFEVTYTRNQDILKNRPGIHYAPPILEKNKDGQRLIVTYEVDWKNKTVKVVDKYSDDNKPYKEG*

**LukB:**

MNSA**EQKLISEEDLGS**KINSEIKQVSEKNLDGDTKMYTRTATTSDSQKNITQSLQFNFLTEPNYDKETVFIKAKGTIGSGLRILDPNGYWNSTLRWPGSYSVSIQNVDDNNNTNVTDFAPKNQDESREVKYTYGYKTGGDFSINRGGLTGNITKESNYSETISYQQPSYRTLLDQSTSHKGVGWKVEAHLINNMGHDHTRQLTNDSDNRTKSEIFSLTRNGNLWAKDNFTPKDKMPVTVSEGFNPEFLAVMSHDKKDKGKSQFVVHYKRSMDEFKIDWNRHGFWGYWSGENHVDKKEEKLSALYEVDWKTHNVKFVKVLNDNEKK*

Key:

**6xHis Tag**

**cMyc Tag**

**Linker**

**Extended data Figure 2: Cytotoxicity and ELISA data for full CC8 LukAB alanine screen on CD18/HVCN1 CHO**

Refer to excel file

**Extended data Figure 3: Negative stain micrographs and 2D classes of WT and V113A LukAB**

Refer to PDF

**Extended data Figure 4: Peptide oxidation of CC8 WT, V113A and K120A LukAB**

Refer to excel file
