# Supplementary material for: Unlatching of the stem domains in the Staphylococcus aureus pore-forming leukocidin LukAB influences toxin oligomerization
Source: J Biol Chem. 2023 Oct 4;299(12):105321. doi: 10.1016/j.jbc.2023.105321 (PMC10665946; doi:10.1016/j.jbc.2023.105321)
Supplement: Extended data Figure 3 [file mmc3.docx]

**Extended data Figure 3: Negative stain micrographs and 2D classes of WT and V113A LukAB**

A	WT, 0% MPD

B	WT, 20% MPD

C	WT, 40% MPD

D	V113A, 0% MPD

E	V113A, 20% MPD

F	V113A, 40% MPD

A-C: Representative micrograph of CC8 WT LukAB in 0% MPD (A), 20% MPD (B) and 40% MPD (C). 2D classes of 7,715 particles for WT LukAB in 40% MPD autopicked from 51 micrographs. Scale bar is 100 Å.

D-F: Representative micrograph (left) and 2D class averages (right) of V113A LukAB in 0% MPD (D), 20% MPD (E) and 40% MPD (F). 2D classes represent 5,755 (D), 6,959 (E) and 13,951 (F) particles autopicked from 51 micrographs. Scale bar is 100 Å.
